# Barriers and facilitators to diabetes screening and prevention after a pregnancy complicated by gestational diabetes

**DOI:** 10.1371/journal.pone.0277330

**Published:** 2022-11-18

**Authors:** Drishti D. Sinha, Roxann C. Williams, Laura N. Hollar, Hannah R. Lucas, Bethany Johnson-Javois, Heidi B. Miller, Amanda Stoermer, Graham A. Colditz, Aimee S. James, Cynthia J. Herrick

**Affiliations:** 1 Division of Endocrinology, Department of Medicine, Metabolism and Lipid Research, Washington University School of Medicine, St. Louis, Missouri, United States of America; 2 Heritage Medical Associates, Nashville, Tennessee, United States of America; 3 St. Louis Integrated Health Network, St. Louis, Missouri, United States of America; 4 Division of Public Health Sciences, Department of Surgery, Washington University School of Medicine, St. Louis, Missouri, United States of America; PLOS ONE, UNITED KINGDOM

## Abstract

**Objective:**

Gestational diabetes mellitus (GDM) is increasing in the United States, with higher rates among minoritized racial and ethnic populations and lower income populations. GDM increases risk for type 2 diabetes (T2DM), and postpartum diabetes screening and prevention are imperative. This qualitative study examines barriers and facilitators to postpartum T2DM screening and prevention among non-privately insured individuals with a history of GDM in a state prior to Medicaid expansion.

**Methods:**

Thirty-six non-privately insured women with a history of GDM completed semi-structured interviews. Four focus groups and seven interviews were conducted with 30 nurse practitioners, physicians, physician assistants, nurses and registered dietitians from Federally Qualified Health Centers in St. Louis, MO. Interviews and focus groups were audio-recorded and transcribed. Data were analyzed using an integrative thematic analysis informed by the socio-ecological model.

**Results:**

Barriers and facilitators to T2DM screening and prevention occur across multiple environments (society, healthcare system, interpersonal, and individual). Societal barriers include insurance issues, unemployment, and lack of transportation, childcare, safe housing, and healthy food access, while facilitators include government sponsored programs and community organizations. Healthcare system barriers include care fragmentation, scheduling policies and time constraints while facilitators include care coordination, pregnancy support groups, and education materials. Interpersonal barriers include negative care experiences, cultural differences, communication challenges, competing priorities, and lack of a social support network, while facilitators include family and friend support and positive care experiences. Individual barriers include health complications and unhealthy food and exercise patterns, while facilitators include child wellbeing, empowered attitudes and healthy food and exercise patterns.

**Conclusions:**

The socioecological model highlights the societal and systemic determinants that encompass individual and interpersonal factors affecting postpartum T2DM screening and prevention. This framework can inform multi-level interventions to increase postpartum T2DM screening and prevention in this high-risk population, including policy changes to alleviate higher-level barriers.

## Introduction

Gestational diabetes mellitus (GDM) affects approximately 6% of pregnancies in the United States [1]. Minoritized racial and ethnic populations and those who are socioeconomically disadvantaged exhibit an even higher prevalence of GDM [2]. GDM is a significant risk factor for the development of Type 2 Diabetes (T2DM) later in life, with over half of patients eventually developing the disease [3], and T2DM may subsequently lead to multiple macrovascular and microvascular comorbidities over a lifetime [4]. Lifestyle interventions and metformin decrease T2DM risk by 50% in women with a history of GDM [5]. Therefore, early and consistent screening and prevention strategies for T2DM (diet, physical activity, breastfeeding, and metformin use) provide an opportunity to mitigate risks. The American Diabetes Association (ADA) and the American College of Obstetricians and Gynecologists (ACOG) recommend T2DM screening at 4–12 weeks postpartum for people previously diagnosed with GDM and every 1–3 years thereafter [6, 7]. However, these guidelines are not universally followed, and disparities in screening are evident for minoritized racial and ethnic populations and patients with lower incomes.

In Missouri, prior to Medicaid expansion, women on Medicaid during pregnancy often lost health coverage after sixty days postpartum [8]. To illustrate the challenges at this transition of care, a study linking Medicaid claims data with electronic health record (EHR) data from Federally Qualified Health Centers (FQHCs) across Missouri found less than 10% of patients received a recommended T2DM screening test within 12 weeks postpartum. Moreover, less than 20% were screened at any point during the first postpartum year [9].

Prior studies have identified barriers and facilitators to postpartum care among patients with GDM. Access to public transportation and diabetes education during pregnancy were significantly associated with recommended postpartum screening among Missouri women receiving care in FQHCs [10]. Common barriers in qualitative studies were fragmented care, insufficient information, challenges of a new maternal role, lack of support in the postpartum period, and inaccurate perception of future T2DM risk [11, 12]. Facilitators of care-seeking included availability of childcare, connection with clinical staff, and social support [11, 13]. Most of these studies were conducted with privately insured participants. In a study of low-income women with GDM, patients discussed negative experiences with providers, loss of healthcare coverage after delivery, and lack of culturally sensitive practices [14]. Only one study conducted in an urban safety-net setting incorporated both patient and healthcare provider perspectives, finding that providers tend to focus primarily on the health of the baby after delivery, and lack of coordination and consistent communication can impede care [15]. A systematic review of qualitative studies examining women’s views on postpartum T2DM screening after GDM diagnosis identified healthcare system, interpersonal and individual level factors that influenced uptake of screening [16].

To address gaps in the literature, this study aims to compare the perspectives of non-privately insured patients previously diagnosed with GDM to those of a wide array of healthcare providers (HCP) who provide care for this population in FQHCs. Understanding how patients and HCP perceive barriers and facilitators to T2DM screening and prevention is important to developing successful multi-level implementation strategies to improve care. In addition to physicians, nurse practitioners and physician assistants, this study included registered dietitians and nurses, whose perspectives have not been incorporated in prior qualitative studies of GDM. The current study also incorporates broader social, cultural, and political factors that affected the healthcare system and patient care access in FQHCs prior to Medicaid expansion in Missouri.

## Methods

Study participants included patients and healthcare providers (HCP). Patients were recruited from the St. Louis Integrated Health Network’s FQHCs and community organizations. Additionally, advertisements were placed on the local radio station (WWHL FM 104.1), public transportation (MetroLink, MetroBus), and the delivery floor of Barnes Jewish Hospital in St. Louis, a tertiary care center.

Eligibility criteria for patients included women aged 18–40 who were affected by GDM during a pregnancy in the past 10 years, as defined by Carpenter and Coustan criteria [17] or physician chart documentation. Ten years was selected because this is the time frame during which women are at highest risk for progression to T2DM. Patients also had to have either Medicaid, Gateway to Better Health (safety-net coverage available to St. Louis residents), an Affordable Care Act marketplace plan (not employer based), or no insurance at the time of the interview. At the time of pregnancy and delivery, all recruited participants had Medicaid. Eligibility was confirmed through medical records.

Patients were stratified by time since GDM diagnosis (<1 year or ≥1 year) and postpartum T2DM screening status (screened vs. unscreened) to ensure representation in the sample. Patients were considered “screened” if they received a recommended test within one year of delivery (i.e. an oral glucose tolerance test or fasting glucose test 4–12 weeks postpartum or an A1c from 12 weeks to one year postpartum). Individual, in-depth, semi-structured interviews were conducted with 36 eligible patient participants in a private room on the Washington University campus. Parking and public transit were readily accessible to the interview location. Patients also completed a demographic survey including age, race/ethnicity, number of pregnancies, insurance status, and highest level of education completed. Participants received free parking or a voucher for public transit, childcare on site if desired, and $50 cash compensation for their time.

Thirty HCP (physicians, nurses, nurse practitioners, physician assistants, and registered dietitians) were recruited from FQHCs. HCP interviewed for this study were not necessarily providing care for the patient participants interviewed for this study. The different types of HCP were recruited in order to incorporate perspectives across the spectrum of individuals who care for patients with gestational diabetes. Four focus groups were conducted with 7 nurse practitioners, 11 physicians, and 3 physician assistants (group 1: women’s health nurse practitioners (n = 5); group 2: family practice physicians and nurse practitioners (n = 5); group 3: OB/Gyn physicians (n = 6); group 4: internal medicine and family practice physicians and physician’s assistants (n = 5)). Seven in-depth, semi-structured interviews were conducted with 5 nurses and 4 registered dietitians (5 interviews were conducted individually; 2 nurses were interviewed together; 2 dietitians were interviewed together). HCP completed a demographics survey including age, race/ethnicity, healthcare role, and years in practice. HCP received a meal at the time of the focus group or interview, which was completed during the normal workday.

Two members of the research team (PI, research coordinator) conducted each interview and focus group with patient and HCP participants. Integrated Health Network leadership and the Generate Health ‘Making Change Happen’ Leaders group provided input during the creation of the interview guide, ensuring community participation and cultural sensitivity. Interview guides for patients and HCP covered similar topics (S1 Appendix). This report focuses on barriers and facilitators to postpartum T2DM screening and prevention. Patient understanding and HCP education about GDM diagnosis, diet and exercise, self-monitoring and medications, T2DM screening and prevention are reported separately [18].

Interviews and focus groups were audio recorded and transcribed. A codebook of themes included code definitions, inclusion/exclusion criteria, and illustrative quotes. Initially, each coder reviewed 3–5 transcripts to identify themes, with subsequent discussion and agreement on the content of the codebook. Two independent raters coded each transcript in NVivo 12 (QSR international) (patients: RCW, LNH; HCP: RCW, HL). There was > 80% agreement before discussion between the two raters, and discussion resolved remaining discrepant codes. A third rater (CJH) was available for discussion when one of the two initial raters (LNH) was no longer available to reconcile codes.

The Washington University Human Research Protection Office approved the study on June 6, 2016. All participants provided written informed consent. Interviews and focus groups occurred from May 2017 through April 2018.

## Results

### Demographics

Participant demographics are outlined in Table 1. Among patients (n *=* 36), 50% were screened for T2DM postpartum. At the time of consent, 47% were less than one year postpartum. Median age at interview was 30.5 years (IQR 26.8–35.3 years), 30.6% were college graduates, and the majority (72.2%) identified as Black or African American. Among HCP (n = 30), 93% identified as female, and the majority (86.7%) were white. When stratified by specialty, 46.7% worked in OB/GYN, 26.7% worked in Family Medicine, and 13.3% worked in Internal Medicine. By degree, 36.7% were physicians (MD or DO), 23.3% were nurse practitioners (NP), 16.7% were nurses (RN/BSN/LPN), 13.3% were registered dietitians, and 10.0% were physician assistants (PA).

**Table 1:** Qualitative interview and focus group participant demographic characteristics.

Women with history of gestational diabetes (n = 36)	
	**Median (IQR)**
Age at Consent	30.5 (26.8–35.3)
# of Live Births	3 (2–4)
	**n (%)**
Screening Status	
Screened	18 (50)
Unscreened	18 (50)
Time Since Delivery	
< 1 year	17 (47.2)
≥ 1 year	19 (52.8)
College Graduate	11 (30.6)
Race	
Asian/Another race/More than one race	4 (11.1)
Black or African American	26 (72.2)
White	5 (13.9)
Unknown	1 (2.8)
Ethnicity	
Hispanic, Latina, or Spanish Origin	2 (5.6)
**Healthcare Providers (n = 30)**	
	**n (%)**
Specialty	
OB/Gyn	14 (46.7)
Family Practice	8 (26.7)
Internal Medicine	4 (13.3)
Not applicable^a^	4 (13.3)
Degree	
MD/DO	11(36.7)
NP	7 (23.3)
PA	3 (10.0)
RN/BSN/LPN	5 (16.7)
RD	4 (13.3)
Years in Practice	
0–9	18 (60.0)
10+	12 (40.0)
Race	
Asian/Another race/More than one race	1 (3.3)
Black or African American	3 (10.0)
White	26 (86.7)
Female	28 (93.3)
Male	2 (6.7)

^a^ Dietitians were not located within a particular specialty department

The coded data coalesced around themes of barriers and facilitators at the societal level, healthcare system level, interpersonal level (reflecting relationships between HCP, patients and their families) and individual level (reflecting challenges and supports from the patient perspective only). These themes are discussed below with exemplifying quotes in designated tables and additional quotes in supporting information (S2 Appendix). In Tables 2–4, when patient and HCP participant perspectives are presented for a subtheme, the patient perspective is presented first, followed by the HCP perspective. HCP participants have HCP included in their participant number. Table 5 includes patient participant perspectives only.

**Table 2:** Societal level barriers and facilitators to postpartum diabetes screening and prevention: Exemplar quotes.

Barriers	
**Sub-theme**	**Participant perspectives**
**Insurance issues**	Because after I moved I didn’t have health insurance for a while and then I got Medicaid and, you know, only so many people take Medicaid in certain cities than a lot of those places are just completely bombarded with patients so they can’t see you immediately (participant 5, unscreened, delivered ≥ 1 year).
	If we have patients sometimes that do not have Medicaid…Even though they are pregnant they do not qualify you know they are undocumented or they did not send in a certain paper…they are temporary expired…sometimes getting their supplies is a challenge…our pharmacy here will not dispense even with the prescription if they don’t have Medicaid (participant 23HCP, Ob/Gyn, Nurse).
**Employment Struggles**	When I was financially able, I didn’t have the time to go…And then when I have the time, I wasn’t financially able to take off (participant 50, unscreened, delivered ≥ 1 year).
	If they have any sick benefits, they’ve probably used them up during their pregnancy or in that those early weeks…if they work nights, that’s difficult because they don’t want to go to an appointment in the morning or…after they get off work…when they could be sleeping…schedules are a huge barrier (participant 31HCP, registered dietitian).
**Housing Instability**	We did end up being homeless at one point so I had to move in with someone so it was just a lot going on that I, I was not thinking about health or anything [laughs] (participant 25, screened, delivered ≥ 1 year).
	St. Louis is not exactly a safe city… I encourage all my patients to try to get a 20-minute walk every day but then you know they live in an area that I would not even walk in. Why would I encourage them to walk for 20 minutes…it’s just the reality of life here (participant 6HCP, Ob/Gyn, Nurse Practitioner).
**Food insecurity**	When they suggest new things for you to eat, like I said…I was pregnant, I am single mother, I have a kid and I didn’t have the money to…buy healthy. So they did give us WIC… Which was like a fruits and milk but it was not enough for main course meals. . .I do not have enough money to do all of that you know (participant 32, unscreened, delivered ≥ 1 year).
	I think that the biggest barrier when I do education initially, and people are like oh my gosh you know in my neighborhood how am I going to find the healthy food that you’re trying to tell me I’m supposed to eat (participant 3HCP, Ob/Gyn, Nurse Practitioner).
**Transportation challenges**	When you’re a single parent and you don’t have a car, you do not have transportation, trying to get somewhere with a baby…you don’t like to take your newborns out (participant 46, screened, delivered ≥ 1 year).
	North city doesn’t have any coverage on bus stops anywhere… So if the weather is bad, you’re sitting out there with you and your kids and there’s no covering at the bus stop (participant 11HCP, Ob/Gyn, Physician).
**Lack of Childcare**	I had a 1-year-old and a newborn.. it’s hard to find childcare if you want to go to the doctor because some Dr’s offices don’t allow you to bring children so that was a huge issue too (participant 5, unscreened, delivered ≥ 1 year).
**Facilitators**	
**Sub-theme**	**Participant perspectives**
**Community organizations**	I started using…my resources with food pantries… because they always have vegetables and stuff to give away, even if a few of them maybe a little, you know…Suspect [laughs]. But you still [laughs] supplement income like that (participant 31, screened, delivered ≥ 1 year).
	I think they rely on Nurses for Newborns. To do that, those home visits for the pregnant and … right after delivery patients. I mean there’s Parents as Teachers. But they don’t really … know … this mom has gestational diabetes. So we are going to follow her and go do all visits. I don’t think they really do that. They just, I think they just don’t have the staff (participant 23HCP, ObGyn, Nurse).
**Government sponsored programs**	I have Medicaid so it, it takes care of everything (participant 13, unscreened, delivered < 1 year).
	It would be beneficial for WIC to have access to that to be like your appointment’s next week or you missed your appointment yesterday….They definitely come back WIC. They don’t miss that. Even without insurance (participant 4HCP, Ob/Gyn, Nurse Practitioner).

**Table 3:** Healthcare system level barriers and facilitators to postpartum diabetes screening and prevention: Exemplar quotes.

Barriers	
Sub-theme	Participant perspectives
**Care fragmentation**	Say for instance I got a doctor’s appointment while my baby got a doctor’s appointment…Then doctor’s appointment takes at least an hour time to almost 2. It takes almost 30 minutes to even get registered and for them to call you back…Just to get you…prepped…Okay, my appointment was for 12. I get out of here about almost 2. Who wants to sit over there and take another hour test (participant 44, unscreened, delivered < 1 year)?
	They [WIC] actually have a separate EHR that we can’t even see their notes…So it’s really on behalf of the nutritionist who is worried about somebody that they might proactively reach out to us (participant 9HCP, Family Practice, Physician.)
**Scheduling policies**	Our no-show policy…says that if a patient has missed 3 or more appointments in the past 12 months that…they become suspended which means they go through the 6 months period where they cannot obtain any scheduled appointments…Unless they’re specifically given permission by the doctor (participant 25HCP, Family Practice, Nurse).
**Appointment time constraints and staffing limitations/needs**	I just didn’t like the high risk clinic. That’s all. They take forever. They have you there for hours and hours and hours. And especially when they only see you for like 5 minutes (participant 4, screened, delivered ≥ 1 year).
	To me, the best thing would be if we could have that diabetic educator right there in the clinic that as soon as they finish with us, they can go right to that person (participant 20HCP, Family Practice, Physician Assistant).
**Facilitators**	
**Sub-theme**	**Participant perspectives**
**Care coordination**	I was for the morning because of the fasting. I didn’t want to go, you know, to have the appointment in the afternoon (participant 2, screened, delivered ≥ 1 year).
	I feel like it’s more than just giving the patient a prescription and sending them out the door… if you’re going to care for a patient’s wellbeing, that includes everything…what they have to eat and how they obtain that food and, you know, is their home life safe and do they have a roof over their heads and… we can’t put a Band-Aid on diabetes (participant 25HCP, Family Practice, Nurse).
**Pregnancy support groups**	I really felt like that was a good class because it…is a group for moms… we are all in there together and just sharing our experiences, throughout our pregnancy together…And helping each other get through the pregnancy … it’s like the class became a family (participant 1, screened, delivered ≥ 1 year).
**Patient education materials**	Just one general website that has different links, different journals or reviews whatever. Like just one main location instead of having you know things everywhere (participant 45, screened, delivered < 1 year).
	We have several different handouts that we pulled from hospital resources online or just ordered from 3 nutrition organizations with evidence-based diabetic diets. We also have some WIC resources too that are gestational diabetes based, we also had some sample menus from other dietitians that were from other hospitals that I might use sometimes (participant 29HCP, Registered Dietitian).

**Table 4:** Interpersonal level barriers and facilitators to postpartum diabetes screening and prevention: Exemplar quotes.

Barriers		
Sub-theme	Participant perspectives	
**Negative experiences with HCP**	Because I have a couple of doctors where I didn’t feel that they…were sympathetic or compassion towards patients… At all, so I feel like if you don’t have any sympathy or any compassion, you really can’t be a doctor or work with people in general… Because you can’t see them as your own like this is my child… putting yourself in someone else’s shoes on how you wanted to be treated (participant 12, screened, delivered ≥ 1 year).	
	[In] one of those publications…they interviewed people in some of the zip codes that we serve, some of the more impoverished zip codes and…many of the people interviewed were not only dissatisfied with the care being provided at the health centers. There were complaints about wait time and physician communication…the general distrust of the healthcare community… the sense that [we] were profiting off of them …And I don’t know if that extends back to some of the distrust of medications and then people feel like well I want to find my own way… with diet or herbal things (participant 20HCP, Family Practice, Physician Assistant).	
**Cultural differences**	Like for instance with the Latino population. A lot of the traditional foods are very carb heavy and so just trying to be flexible and careful on how you educate and counsel and help them come up with ways to make substitutions. I think that can be a barrier too for some people maybe to feel like they’re being stripped of their culture when it comes to just learning about they’re eating more of a diabetes friendly diet. In every culture, it’s a big thing and so for them to not feel like they are missing out. We’re just giving them good strategies and good tips to make things work without them feeling deprived (participant 29HCP, Registered Dietitian).	
**Communication challenges**	I be trying to tell them they got to use like small words [laughs]. They using them doctor words and stuff. . .And I just be like okay…switch it up a little bit…But they don’t, they still saying the big words [laughs]. I don’t understand all that doctor talk (participant 42, unscreened, delivered < 1 year).	
	We have a large population of non-English speaking patients and our interpreting services are usually either telephonic or video…there is only so much you can get out of that. You don’t have the body language. You don’t have that rapport (participant 25HCP, Family Practice, Nurse).	
**Lack of family or friend support**	I think that was the hardest part and it still is and…when you have kids, it’s hard. Because even if you try to follow a healthy diet, the kids don’t want to eat what you’re eating so it’s extra work (participant 50, unscreened, delivered ≥ 1 year).	
	I think one other barrier can be just lack of support from the family or just lack of understanding…also moms that have many other kids and then have to manage the illness on their own (participant 29HCP, Registered Dietitian).	
**Competing priorities**	I had bigger things to worry about at the time. And I couldn’t really focus on, you know, I wanted to do those things but I have 4 kids so [laughs] it was like I have to …Juggle everything at once. It’s very hard to do (participant 10, unscreened, delivered ≥ 1 year)	
	I just see that a lot like…With mom doing things for their kids but not for themselves (participant 27HCP, Ob/Gyn, Nurse).	
**Facilitators**		
**Sub-theme**		**Participant perspectives**
**Positive experiences with HCP**		I had her number on call, like if I really, really need her, so she kind of talked to me on a woman to woman level and not on a medical level. For me to see how concerned she was about my health, it really just gave me the inspiration to take it serious too (participant 2, screened, delivered ≥ 1 year).
**Family or friend support**		My children are my biggest supporters. They really are and then like I say my doctor [laughs]. Those are my main number one fans. That’s kind of like yeah I want better for them, you know, and they can’t do it their selves… Then when they get older, they can teach their children what I taught them (participant 30, unscreened, delivered < 1 year).

**Table 5:** Individual (patient) level barriers and facilitators to postpartum diabetes screening and prevention: Exemplar quotes.

Barriers		
Sub-theme	Participant perspectives	
**Infant health and delivery complications**	One of the most important things was to get my son home [from the NICU]…nothing else mattered (participant 44, unscreened, delivered < 1 year).	
**Maternal comorbidities and symptoms**	Seven months, I was almost 200 pounds at that time so…they told me I did need to lose weight…but depression is a really kicker (participant 10, unscreened, delivered ≥ 1 year).	
**Negative emotions**	It is overwhelming in the beginning and then…you’re scared…especially if it is your first child… you don’t know what’s coming on with pregnancy first off so then you throw in the diabetes and it’s like what (participant 3, screened, delivered < 1 year).	
**Inaction with regard to guideline adherence**	It was very important to follow those directions but when you are in denial…you are disobedient, you are not going to follow it (participant 11, unscreened, delivered < 1 year).	
**Unhealthy food and exercise patterns**		Just sitting at home I could exercise…the baby’s not an excuse. I’m not going to say that [laughs] so it’s just an effort thing, you know, just get up and do it and stop saying it and me talking about me wanting to lose weight but sitting there…it’s not going to help me (participant 30, unscreened, delivered < 1 year).
**Facilitators**		
**Sub-theme**		**Participant perspectives**
**Children’s wellbeing**		You want to be able to…not let the disease defeat you. I have a lot to live for. I want to see my boys grow up (participant 32, unscreened, delivered ≥ 1 year).
**Empowered attitudes**		Speak up and ask questions, state your concerns, comments, anything. A lot of people feel like…that person might not know what I’m talking about or they might not understand…if you just pay attention to yourself and your body then and you find the right doctor … you should just speak up about it (participant 36, unscreened, delivered < 1 year).
		Being you know the Black community, and how you know, the mistrust… With doctors. I’m going to tell everybody, you have gestational diabetes, you want to [laughs] go talk to the people… to help (participant 31, screened, delivered ≥ 1 year).
**Healthy food and exercise patterns**		You have to do something that you already like to do is what I found. Like, I can’t just do something that someone is saying oh well do 10 sets of this and 20 reps of this. For me, it was like dancing. It was like walking (participant 53, screened, delivered < 1 year).

### Barriers: Society

Societal barriers include structural issues that systematically prevent non-privately insured patients from engaging in GDM management, T2DM screening, and T2DM prevention (Table 2). Patients and HCP noted similar barriers; however, patients uniquely noted childcare as a prominent barrier.

#### Insurance issues

Patients commonly cited loss of Medicaid after pregnancy, disruptions in care continuity related to insurance changes, and difficulty finding providers who accepted Medicaid as barriers. At the time of the interviews, Missouri had not expanded Medicaid, and HCP highlighted the “cumbersome and obstructionist” (unidentified participant, Ob/Gyn, Focus group 3) process of transitioning from Medicaid to Gateway to Better Health after pregnancy. HCP also noted waiting periods for coverage in early pregnancy affecting prenatal care and lack of coverage for undocumented patients.

#### Employment struggles

A majority of patients who described employment struggles as a barrier to care were not screened for T2DM postpartum. Unemployment was associated with financial struggles and insurance loss, inhibiting the ability to seek care. However, employed patients described challenges related to inflexible work schedules, coordinating multiple jobs, and work environments that did not accommodate mothers (e.g. physical challenges during pregnancy, time for appointments, pumping breastmilk, inadequate maternity leave). HCP underscored that poverty exacerbates the difficult tradeoffs patients face with employment and appointment attendance, highlighting inadequate maternity leave.

#### Housing instability

The majority of patients who cited unfavorable home environments as a barrier to care were unscreened for T2DM postpartum. Patients noted that an unsafe home environment, related to intimate partner violence, lack of sanitation, and pest infestations, supersedes healthcare. Both patients and HCP noted that being homeless or living in temporary housing impedes healthcare seeking. HCP noted housing instability as a barrier to establishing contact for appointment reminders or rescheduling missed appointments. HCP also perceived that unsafe neighborhoods inhibit physical activity recommended for GDM management and T2DM prevention.

#### Food insecurity

Both patients and HCP cited limited access to healthy foods, related to unaffordability or location inaccessibility, as a barrier to both GDM management and T2DM prevention efforts. Government assistance programs, such as the Special Supplemental Nutrition Program for Women, Infants and Children (WIC) (a program that provides healthy food education and supplemental meals to low-income pregnant and postpartum women and their infants) and the Supplemental Nutrition Assistance Program (SNAP), did not always provide adequate support. For example, both patients and HCP highlighted the inclusion of juice as standard in WIC meal packages, which is not appropriate for patients with GDM.

#### Transportation challenges

Lack of convenient transportation was a primary barrier, cited by patients and HCP, to attending postpartum appointments. Medicaid sponsored transportation was often noted to be unreliable, and public transportation or walking could be inconvenient, difficult in poor weather, and at times, unsafe.

#### Lack of childcare

Patients, but not HCP, mentioned lack of childcare as a major barrier to T2DM screening and prevention. Childcare was often unaffordable or unavailable, and it was difficult to focus on maternal health or discuss private health concerns when attending visits with children. Additionally, some patients reported that providers would prohibit children at appointments. Finally, lack of childcare affected diabetes prevention efforts by making other healthy behaviors more difficult to accomplish.

### Facilitators: Society

Patients and HCP noted that community organizations and government programs could mitigate some of the barriers to GDM management, T2DM screening, and T2DM prevention (Table 2).

#### Community organizations

Patients and HCP appreciated community organizations providing home health access, community health access, and healthy lifestyle support. They highlighted programs such as Parents as Teachers (home visits by parents focusing on family centered assessments and goal setting) and Nurses for Newborns (pediatric nurse home visiting to monitor infant health). However, both programs concentrate more on the child than on the mother’s health. Some patients were able to find accessible community fitness organizations and found food pantries helpful to supplement their weekly groceries with fresh produce. Others noted farmer’s markets that accept government assistance funds were a way to support local Black businesses.

#### Government sponsored programs

Both screened and unscreened patients and HCP noted Medicaid as a critical facilitator for healthcare access, supplies, and medication. Despite its limitations, WIC was mentioned as a facilitator by HCP and patients who were screened. Advantages of the program included nutrition advice, literature, recipes, and healthy food options, which made it easier to adopt a healthy diet. WIC also connected patients to other social services. HCP believed that WIC was easier to apply for than Medicaid and that patients had better pregnancy outcomes when WIC assistance started early. Patients also considered other government programs such as unemployment insurance and SNAP to be important societal supports.

### Barriers: Healthcare system

Within the healthcare system, patients highlighted long wait times as primary barriers to care and HCP noted problems with scheduling policies. Both groups mentioned barriers associated with care fragmentation and appointment time constraints (Table 3).

#### Care fragmentation

Several patients mentioned that it was burdensome to have providers at different clinics for mother and child. People also said that long appointment wait times made it impractical to schedule T2DM screening the same day as their babies’ appointments. Patients also highlighted communication issues among HCP that further fragmented care (i.e. providers leaving practice, lapsed communication among specialists).

HCP advocated for coordinating patient and infant appointments, and underscored communication lapses within and among care teams as a challenge. Clinic and hospital system electronic health records (EHRs) do not communicate and patients move between systems, charting systems differed across providers, with incomplete transfer of historical information, and WIC offices housed within FQHCs have separate record systems. EHR notifications for upcoming screenings are inconsistent. Further, there is no clear consensus on who should manage postpartum T2DM screening, and management and prevention of diabetes is scattered across specialties.

#### Scheduling policies

Strict no-show and late policies at clinics compounded societal barriers, such as lack of transportation and difficulty taking time off from work, further inhibiting prevention, follow-up care, and screening for T2DM. Moreover, scheduling systems did not adequately track missed and cancelled appointments in order to facilitate pro-active rescheduling. HCP also noted that postpartum visits were not automatically scheduled if delivery occurred at a hospital unaffiliated with the health center. Scheduling postpartum T2DM screening can also be burdensome because fasting is required.

#### Appointment time constraints and staffing limitations/needs

Patients felt that appointment time constraints coupled with long wait times hindered quality care for GDM and T2DM prevention. Patients recognized that busy health centers were understaffed. However, they felt that appointments were too short for the long wait time, creating a disincentive for appointment attendance. HCP also noted that appointment time constraints prevented relationship building and in-depth education on the risk of developing T2DM. To address time constraints, multiple HCP wanted to have an onsite diabetes educator or dietitian, or individual who could do home visits. Even when present on site, registered dietitians often felt they had insufficient time to provide education.

### Facilitators: Healthcare system

#### Care coordination

When care was coordinated, this often facilitated postpartum T2DM screening and prevention (Table 3). Scheduling six-week postpartum appointments in the morning helped patients with screening because of fasting requirements. HCP noted the importance of having role definition within care teams, onsite lab facilities and follow-up appointments at the health center scheduled at hospital discharge after delivery. HCP emphasized the importance of the “warm handoff,” directly introducing the patient to the next provider, as well as direct communication with patients to schedule follow-up appointments. Moreover, while HCP mentioned EHR limitations, there were also examples of good communication between specialties and health centers.

Patients and HCP recognized the important role of home health nurses and community health workers employed by the health center in facilitating diabetes prevention education, follow-up appointments, and healthcare coverage. HCP noted coordination and co-location of health centers and WIC as tactically important to facilitate appointments and improve patient care.

#### Pregnancy support groups

Health center-sponsored pregnancy support groups provided patients with GDM education, parenting skills, nutrition instruction, and general support. Patients used support groups as an avenue to share their pregnancy experiences, while learning from others’ experiences with GDM. While a majority of patients found these groups to be helpful, some patients would have preferred one-on-one instruction and others found the information overwhelming. Additionally, some patients did not have the opportunity, could not afford, or did not want to attend support groups. Groups were also not routinely continued postpartum.

#### Patient education materials

Standardized patient education materials were helpful, particularly when staff may not have specialized training in GDM. Nutrition, exercise and glucose log information, and professional society guidelines were sometimes available in the EHR. At times, staff used materials provided by pharmaceutical companies or obtained online. While HCP appreciated the wide variety of informational sources, they expressed concern that patients might not read the information. Some nurses adapted information to be more accessible and culturally appropriate but were limited in available language translations. Importantly, WIC has many accessible materials for people across a range of literacy levels, but they have limited resources specific to GDM and limited access to registered dietitians. Patients noted that they enjoyed hands-on visual demonstrations (as with food models) and had varied preferences for electronic vs. written materials. They also reported feeling overwhelmed and preferred a single comprehensive resource.

### Barriers: Interpersonal

Interpersonal barriers stem from relationships, embedded within higher-level themes (Table 4). Patients recounted negative experiences with HCP and had difficulty recalling professional guidance. HCP perceived challenges in interactions with patients to include cultural differences, health illiteracy and innumeracy, language barriers, and competing priorities. Both patients and HCP discussed lack of family or friend support as an interpersonal barrier.

#### Negative experiences with HCP

Of the patients reporting negative experiences with HCP, a majority were unscreened. Some patients reported that HCP were rude or lacked compassion and did not listen to or believe them. Patients also reported that communication from HCP about their diagnosis was unclear. This, in combination with time constraints and other societal and healthcare system barriers may have contributed to these negative experiences, fueling a distrust of HCP and the healthcare system among patients. This distrust was furthered by a perception that doctors were only providing care to make a profit.

#### Cultural differences

Cultural differences between HCP and patients presented challenges when delivering care for management of GDM and prevention and screening for T2DM. Carbohydrate-heavy foods like rice and bread are often staple cultural foods and difficult to replace. Additionally, HCP found different cultural expectations among patients regarding appropriate weight gain in pregnancy challenging to overcome. HCP also found it difficult to convey the importance of identifying pre-symptomatic diabetes if their patients viewed illness as only associated with symptoms. Some HCP comments revealed a lack of appreciation of the cultural significance of diet, particularly regarding changes in diet and fasting for important religious celebrations, such as Ramadan.

#### Communication challenges

Patients highlighted medical jargon used by HCP that was difficult to understand at times. HCP noted that patients sometimes had difficulty with health literacy and numeracy which could be a barrier to T2DM screening and prevention. Language barriers between HCP and patients compounded the difficulty in conversations and building rapport. Additionally, HCP noted limited in-house translation services and highlighted that translation extends appointment times, which intensifies the healthcare system barrier of appointment time constraints.

#### Lack of family or friend support

Not having family or friend support was a major interpersonal barrier to management of GDM and prevention of T2DM reported by patients. Some patients felt that they lacked support from their partners or their infants’ fathers, and some indicated they were in unhealthy or abusive relationships. Patients also felt family and friends did not understand their experience or provided advice that was different from HCP’s advice. Additionally, some families did not seem to support patients in healthy diet and exercise choices. HCP also perceived that many of their patients lacked family support, either through absent partners, overwhelming childcare responsibilities, or underestimation of the seriousness of diabetes.

#### Competing priorities

Finally, patients noted busy schedules, lack of sleep, and prioritizing their children over themselves as sources of competing priorities that hindered postpartum screening and diabetes prevention. HCP also believed that one of the most salient barriers present at the interpersonal level to T2DM prevention and screening was competing priorities such as having a new baby, other children, unstable living situations, and/or comorbidities.

#### Facilitators: Interpersonal

Despite barriers noted above, patients also described positive care experiences with HCP and family or friend support that facilitated T2DM prevention and screening (Table 4). All screened individuals reported these supports, and many unscreened individuals reported them as well. Notably, HCP mentioned few specific interpersonal facilitators.

#### Positive experiences with HCP

Some patients described positive experiences with physicians, nurses, and dietitians, particularly when they felt there was a personal investment in their health and wellness and the relationship was longstanding. These experiences were vital in helping the patient to adopt a healthier lifestyle. A few patients mentioned feeling more comfortable with female physicians, given the personal nature of medical appointments during pregnancy.

Patients often preferred when providers offered alternatives to medication, focused on prevention, and recognized the importance of mental and physical health. Several patients noted positive relationships with healthcare staff, describing them as approachable, hospitable, attentive, comforting, and open-minded. Dietitians, in particular, were a trusted source of advice.

#### Family or friend support

Support systems were a major facilitator in seeking care for GDM and T2DM prevention. Patients’ mothers often had a particularly important role, providing financial support, supporting healthy dietary patterns and diabetes self-management, and providing childcare. Patients also cited support from their fathers, children, extended family, friends, and infants’ fathers. Overall, patients recognized their own personal effort needed to sustain change but attributed much of their success and positive outcomes to support from family and friends, though one patient noted that having too much support and advice can also be overwhelming.

### Barriers: Individual

Individual-level challenges included infant health and delivery complications, maternal comorbidities and physical symptoms, negative emotions, inaction with regard to guideline adherence, and unhealthy food and exercise patterns (Table 5). Given the personal nature of these barriers, only patient data are reported in this theme.

#### Infant health and delivery complications

Patients expressed that it was difficult for them to focus on their own health because of their experiences with delivery and newborn health complications. Almost half of patients reported receiving a cesarean section during delivery, most commonly citing the large size of the infant. Patients described this experience to be traumatic and scary. Afterwards, patients felt unprepared for the recovery process—one patient said, it was the “worst pain of my life” (participant 4, screened, delivered ≥ 1 year). Moreover, poor infant health prevented patients from prioritizing their own health and attending appointments for themselves.

#### Maternal comorbidities and symptoms

The most cited comorbidities included high blood pressure/preeclampsia and postpartum depression with depression complicating adoption of healthier lifestyles. Physical symptoms, including nausea, pain, and fatigue, during and after pregnancy often impeded management of GDM and T2DM prevention.

#### Negative emotions

Patients described feeling devastated, depressed, in denial, and scared when diagnosed with GDM. The diagnosis and comprehension of diabetes education was limited because of the emotional toll of the diagnosis. Guilt over potential effects on the baby, denial, and frustration with management tasks were commonly expressed. Anxiety over the possibility of T2DM was either a barrier (scared of the diagnosis) or a facilitator (desire for reassurance) to screening. A few patients described their eventual diagnosis of T2DM as upsetting because they felt they had done what they were supposed to do to prevent the disease.

#### Inaction with regard to guideline adherence

Patients often acknowledged that they had not to adhered to GDM management, dietary, exercise, and T2DM screening guidelines, either because of denial or lack of appreciation for the seriousness of illness [18]. Some patients did not describe why they chose not to get screened, even while acknowledging its importance.

#### Unhealthy food and exercise patterns

Patients recounted that their unhealthy food patterns prevented them from adopting dietary changes to manage GDM and prevent T2DM. Pregnancy-related cravings were difficult to overcome for patients, particularly for non-recommended foods in GDM, such as sodas, desserts, and breads. Patients also reported struggling with portion control and the effort required to cook in the midst of a busy schedule, often relapsing into unhealthy eating habits and hiding this from their families.

Several patients said that they did not exercise during pregnancy and/or do not currently exercise. Physical discomforts of pregnancy made exercise goals difficult to accomplish. Many patients felt that their current employment provided adequate physical activity, while others felt they did not have the motivation or positive mindset to engage in physical activity.

### Facilitators: Individual

Individual-level assets that facilitated T2DM screening and prevention activities included a concern for children’s wellbeing, empowered attitudes, and healthy food and exercise patterns (Table 5).

#### Concern for children’s wellbeing

Children were often a significant motivation to live a healthier life. During pregnancy, patients were motivated to manage their GDM for the sake of their infants’ health. Postpartum, patients valued the time spent with their children. The desire to be a healthy role model and watch their children grow was an impetus for changing maternal lifestyle habits. Patients also felt motivated to create a healthier environment for their own children to prevent them from developing the same health problems.

#### Empowered attitudes

Patients demonstrated a facilitative, empowered attitude, and identified sources of motivation for change, including family history, personal motivation, future pregnancies, avoiding negative consequences of diabetes, and comorbidities. Belief that T2DM is preventable stemmed from this motivation. A majority of the patients who believed that diabetes was preventable, and that it was important to take early action, were screened. Patients’ empowered attitude also manifested as a desire to help others, through cultural empowerment. Patients emphasized the importance of self-advocacy in healthcare and taking initiative to self-educate, find resources, and ask for help as well as prevent disease in their communities, unlearn misconceptions and dismantle distrust in healthcare.

#### Healthy food and exercise patterns

Some patients adopted healthy food behaviors, limiting sodas, carbohydrate heavy foods, fatty foods, and processed foods in their diets. They also reported meal preparation techniques to assist in cooking homemade meals. Diet changes were motivated by the same factors mentioned above, and patients believed a change in mindset was necessary to work toward a healthier diet. Patients reported several positive outcomes associated with their dietary changes, such as intentionally losing weight, feeling better, and genuinely enjoying healthier foods. Some patients also reported engaging in physical activity, such as dancing, walking, playing with kids, yoga, biking, and staying active at work. Exercise was deemed important to facilitate weight loss and alleviate pain and was easiest to incorporate with childcare and finding activities that were enjoyable and accessible.

## Discussion

Our study elicited perspectives from a diverse group of patients and HCP to better understand the societal, health system, interpersonal, and individual barriers and facilitators to GDM management, postpartum screening and prevention of T2DM in individuals previously diagnosed with GDM. Key points are summarized in Table 6.

**Table 6:** Key points.

This study highlights barriers and facilitators to type 2 diabetes (T2DM) screening and prevention among non-privately insured patients previously diagnosed with gestational diabetes, with perspectives included from patients and a professionally diverse group of healthcare providers.
Participants identified barriers and facilitators at the societal, healthcare system, interpersonal and individual levels of the socioecological model.
Barriers across interpersonal, healthcare system and societal levels, including challenges in communication, care fragmentation, scheduling policies, and time constraints, and insurance, employment and transportation issues, may amplify mistrust in existing systems. Facilitators at all levels involved wellbeing, support and coordination.
This framework identifies the multiple factors that influence postpartum T2DM screening and prevention of T2DM to inform future interventions aiming to address barriers and leverage facilitators through a multi-level approach.

The study population has been historically understudied—non-privately insured, majority Black, patients from a mid-sized Midwestern city in a state that had not yet expanded its Medicaid program. Additionally, the inclusion of individuals who were both within and beyond one year of delivery allows for a better understanding of the factors that affect long-term outcomes beyond the immediate postpartum period. The inclusion of professionally-diverse HCP such as physicians, physician assistants, nurse practitioners, nurses, and registered dietitians uniquely elucidated barriers and facilitators at multiple levels and points of care.

### The socioecological model

**Fig 1:**
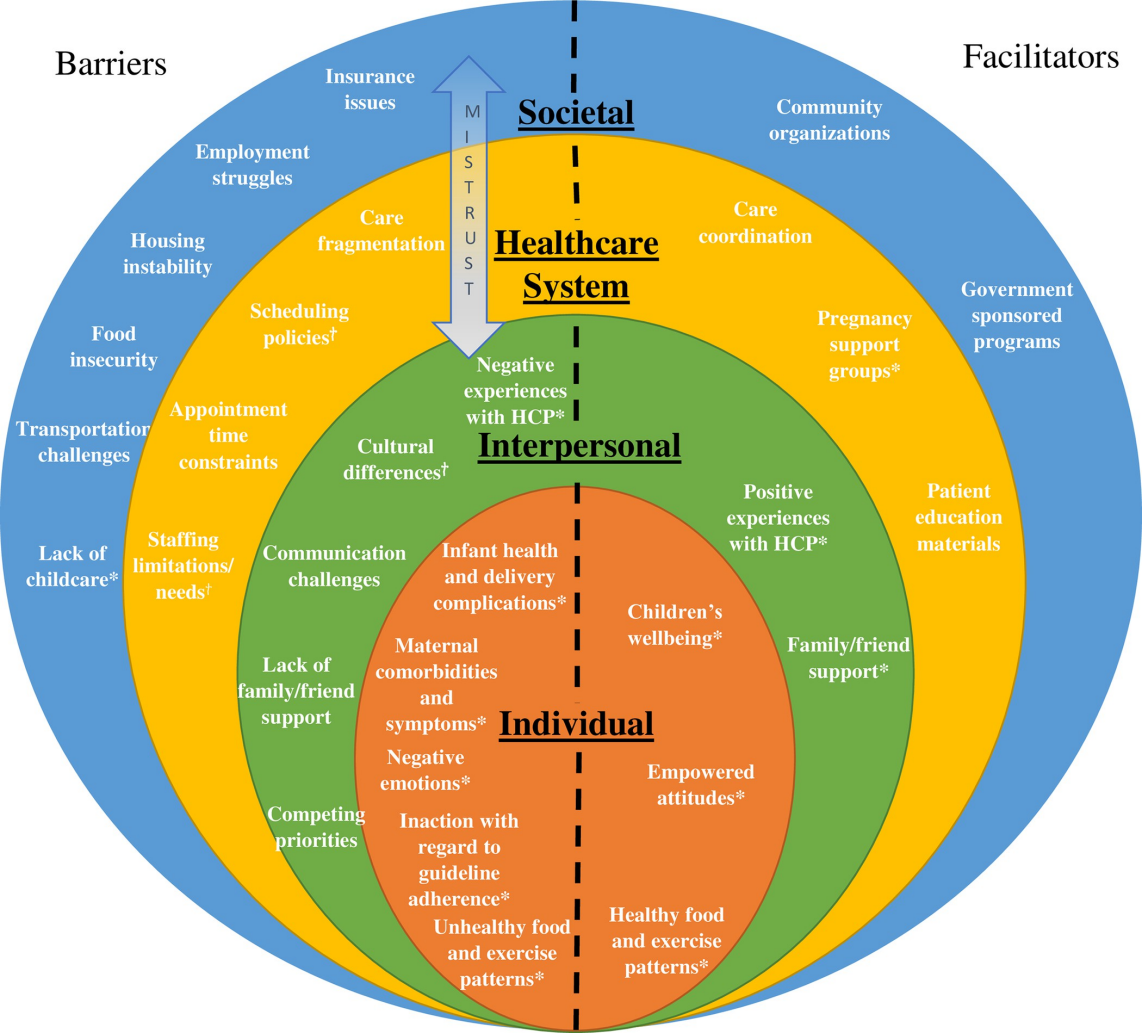
Socioecological model adaptation with barriers and facilitators to T2DM screening and prevention identified by patients and HCP. No symbol: mentioned by HCP and patients. *mentioned by patients only. ⴕmentioned by HCP only.

The socioecological model was applied to our data to illuminate the multiple, nested levels of factors that influence screening for and prevention of T2DM (Fig 1). This differs from prior qualitative studies in this area that have focused primarily on factors at the individual, interpersonal, and health system levels. The socioecological model offers a framework to study health behaviors and ultimately inform behavioral change and public health interventions [19], including determinants of diabetes and pregnancy complications [20–23]. This model emphasizes the interconnectedness of multiple environments and highlights the societal and structural environments as significant influences on the ability to access T2DM screening and prevention.

Per this framework, barriers and facilitators were categorized into societal, healthcare system, interpersonal, and individual levels. Barriers and facilitators mentioned by both HCP and patients were included in the same model to allow for direct comparison between factors highlighted by each group. We have organized the socioecological model of our data from the patient perspective; hence, individual factors were solely those that were identified by patients. HCP perceptions of patient barriers and facilitators are incorporated at the interpersonal level.

Societal barriers were particularly salient for both patients and HCP. One example of this is food insecurity. Patients particularly expressed a desire for healthy food but had difficulty accessing it in their neighborhood. As in many urban areas, lower income neighborhoods in St. Louis often lack access to affordable healthy foods related to multifactorial causes such as a history of red-lining and segregation as well as lack of profitability for food retailers [24].

The nested nature of the model underscores the relationships among the societal, healthcare system, interpersonal, and individual environments. For example, lack of transportation may lead to missed appointments due to appointment time constraints and inflexible scheduling policies, which can contribute to patients’ negative experiences with HCP, resulting in non-adherence to guidelines, and ultimately contributing to societal distrust in medicine. Similarly, facilitators at the societal level, such as expanded Medicaid coverage for pregnant women can improve access to care. When combined with healthcare system facilitators, such as availability of trained HCP, this contributes to positive care experiences and healthier individual behaviors, fostering empowered attitudes among patients. Finally, language barriers, along with lower health literacy and numeracy, are interpersonal barriers nested within the healthcare system which does not have sufficient translation services and adapted education tools to accommodate all patients.

### Differences between patient and HCP perspectives

While many reported barriers and facilitators to T2DM screening and prevention were similar among HCP and patients, differences were noted at every level of the socioecological model. At the societal level, HCP did not identify the lack of childcare as a significant barrier to screening and prevention but this was a prominent barrier identified by patients and discussed in the wider context of other societal barriers, such as lack of employment leading to unaffordability or unavailability of childcare. Patients also perceived that HCP contributed to this barrier by discouraging children at appointments. Patients alluded to societal distrust in healthcare, particularly in discussions of negative experiences with HCP. HCP explicitly identified this distrust as a barrier to care, and importantly, HCP did not blame patients for distrust but rather expressed understanding of the systemic etiologies of this distrust.

At the healthcare system level, HCP identified barriers and facilitators that were focused on operational factors, while patients focused on their personal experiences with the healthcare system. For example, while both groups discussed care fragmentation as a major barrier, patients focused more on the personal difficulties of interacting with several points of healthcare, whereas HCP focused on care fragmentation between specialties, within the EHR system, and within the context of losing insurance. Similarly, HCP demonstrated awareness of the ways in which healthcare system policies amplify societal and interpersonal barriers, such as unforgiving scheduling policies. While HCP thought that scheduling T2DM screening on the same day as pediatrics appointments would be helpful, some patients disagreed. Patients also raised the importance of pregnancy support groups as a healthcare system facilitator, which was largely absent from discussion with HCP, indicating the potential to improve HCP awareness of these resources.

Interpersonal factors expressed by patients focused primarily on the personal relationship between the HCP and the patient themselves, while HCP primarily focused on differences between themselves and patients. For example, patients recounted the personality traits of their providers as well as the way they felt during visits. HCP commonly recounted instances of cultural and language differences between themselves and their patients, with some emphasizing cultural sensitivity and others lacking cultural awareness.

Overall, patients identified barriers and facilitators to screening and prevention in the context of their personal experiences while HCP brought attention to more systemic barriers associated with their professional experience, likely in part reflecting the focus of the interview questions.

### Differences between screened and unscreened patients

While several facilitators and barriers to T2DM screening and prevention were shared among both screened and unscreened patients, there were some factors that were predominantly discussed by one group. Screened individuals emphasized specific facilitators to screening and prevention—such as WIC, timing of the postpartum screening, and beliefs that diabetes is preventable and screening is important. Unscreened patients emphasized certain barriers more than screened patients, including employment struggles, unfavorable home environments, negative care experiences, and inadequate information received about screening. While these factors likely are not the sole, driving determinants for postpartum T2DM screening, it may be useful to pay special attention to emphasizing these facilitators and addressing these barriers with interventions.

### Unique contributions to the literature

Certain factors identified here were not previously highlighted in existing literature. These include barriers associated with government-sponsored programs such as WIC and Medicaid and societal distrust in medicine. By including registered dietitians in the sample of HCP, we demonstrated barriers within the WIC system. Patients and HCP described WIC to be less useful because of the inability to make substitutions in meal baskets and encouraging consumption of foods that are not consistent with a carbohydrate restricted diet (i.e., juices, milk, and bread). Specific to Missouri, which was a Medicaid non-expansion state at the time of these interviews, HCP emphasized the discontinuity of care associated with patients losing Medicaid coverage postpartum. Finally, societal distrust in medicine as a system was raised—including distrust in medications, HCP, and the healthcare establishment, likely associated with historical trauma experienced by Black and low-income patients in the healthcare system. Patients alluded to this distrust within their communities and discussed this in the context of their own negative healthcare experiences.

### Common themes with prior literature

The current study also reflected themes found in prior qualitative research. Prior studies have found that lack of childcare was a barrier to screening for and preventing T2DM [11–13, 16, 25]. Prior studies also noted financial strain as a prominent barrier [13, 14, 26–28], a factor that was present at the core of several sub-themes in the current study, such as lack of employment, lack of insurance, transportation challenges, and food insecurity.

Prior studies have also highlighted care fragmentation and communication issues among HCP, and between HCP and patients, particularly in terms of communication through the EHR [12, 14, 15, 29–31]. The present study was able to explore this further by including perspectives from registered dietitians, who highlighted a lack of integration of the health and social services systems (different EHRs for health centers and WIC even when proximally located). Another aspect of care fragmentation noted in prior work is the lack of clear roles and responsibilities—particularly, designating a clear role to track and follow-up with T2DM screening [15]. Finally, themes related to appointment time constraints, timing of postpartum screening, and scheduling policies were also reflected in prior literature [11, 15, 16, 29, 30].

This study and prior work reflect the important function of family and friend support [13, 16, 25, 26, 31]. While family support facilitated healthier lifestyles, follow-up appointment attendance, and T2DM screening, family perspectives can be a barrier if they do not align with medical recommendations or patient goals, and it is important to educate families with patients [31]. A particular barrier mentioned only by HCP in our study was cultural differences, with a predominant sub-theme of culturally appropriate diets. A Toronto, Ontario-based study also highlighted patients’ difficulty adhering to prescribed diets which lacked cultural awareness [26].

Several studies have reported lack of patient motivation as a major barrier to T2DM prevention [16, 27, 28, 30]. In the current study, some HCP also believed that patient motivation was the most important determinant for T2DM screening. This perspective may be problematic if HCP do not also account for higher level (i.e., societal, healthcare system) barriers and facilitators that influence outcomes.

Finally, prior work emphasized the interpersonal level barrier of competing priorities, particularly with family and baby taking precedence over the patient’s health [11–13, 15, 16, 26–31]. The current study echoes the finding in Parsons et al. that patients prioritize their health during pregnancy with strong motivation to keep the baby healthy. Postpartum, the focus remains on the baby’s health and maternal health becomes secondary. Delivery and neonatal complications further lower maternal prioritization of her own health [31]. However, we also found that mothers were empowered to be good role models for their children, so harnessing this motivation may be a key to diabetes prevention.

### Lessons for multi-level interventions

The socioecological model can inform a multi-level intervention that addresses the governmental and healthcare system policies, social norms, interpersonal relationships, and personal motivations that profoundly impact health behaviors and choices [19]. Interventions to increase T2DM screening among patients with GDM must recognize and prioritize social determinants of health. Policy advocacy is needed to better adapt social programs to individual health needs. For example, the recent passage of Medicaid Expansion in Missouri would expand care to an estimated 231,000 Missourians [32], relieving some barriers associated with financial constraints and care fragmentation postpartum once implemented. While policy change takes time, social determinants of health can be addressed through the interpersonal and healthcare levels as well. For example, community health workers have been used to bridge care for patients with diabetes [33], and these individuals could potentially work around the specific social determinants of health preventing their patients from accessing screening and longer term T2DM prevention. Within the healthcare system, interoperable communication systems can facilitate better care coordination, especially for patients without consistent care providers. Relief of appointment time constraints through task-shifting, non-traditional appointment times, and alternatives to in-person appointments (ex. video call, email, phone call) [34] may allow more in-depth patient education and time to address and mend patients’ distrust of the healthcare system. Finally, at the interpersonal level, cultural sensitivity and awareness training, especially in the context of GDM management and T2DM prevention, could further bolster trust in the healthcare system and support culturally-appropriate and health-beneficial behaviors.

### Limitations

Like most qualitative work, this study is limited in its generalizability. Although several barriers and facilitators overlapped with those identified in prior studies with diverse settings, several of these factors are interpreted within the socio-politico-economic context of St. Louis, MO. While the study exhibited the unique voices and experiences of patients and HCP who are not commonly represented in the literature, these perspectives are not wholly representative of the populations from which these samples were drawn. It is notable that the sample of HCP may be especially prone to selection bias, given they were recruited from only two FQHCs, while patients were recruited from several community settings. Our sample size was sufficient to reach saturation of themes as per qualitative study best practices; however, given the limited number of participants and the wide range of experiences, it is not possible to stratify the groups, for example, by patient education or HCP role, to formally compare and contrast responses. Our qualitative study intended to explore common barriers and facilitators to care and identify potential opportunities for intervention.

Given the dynamics of qualitative interviewing, some interviewers were not the same race/ethnicity or socioeconomic position as patients, who were majority Black and low-income. The intersectionality of racial/ethnic, economic and education differences between interviewers and participants could potentially affect participants’ storytelling related to power differences, discomfort, and perception of the interviewer as an outsider, mistrust of the interviewer, and code-switching as a result of racial trauma [35, 36]. Related to these differences, interviewers and patients may have been deterred from delving into cultural and racial determinants of T2DM screening and prevention. Additionally, there was racial/ethnic discordance between HCP and patient participants in this study. The majority white sample of HCP may not have been as attuned to the social and structural determinants underlying disparities in screening and prevention of T2DM specific to Black patients, and these barriers and facilitators may not have been fully explored.

## Conclusions

Perspectives from non-privately insured patients previously diagnosed with GDM and professionally-diverse HCP examined barriers and facilitators to postpartum T2DM screening and prevention that spanned societal, healthcare system, interpersonal, and individual environments and emphasized the effects of social context on individual health behavior and outcomes. These findings, framed within the socioecological model, will inform multilevel interventions to address barriers and facilitate equitable T2DM screening and prevention in this population.

## Supporting information

S1 Appendix Interview guides. (DOCX)

S2 Appendix Additional exemplar quotes. (DOCX)
