# Supplementary material for: Barriers and facilitators to diabetes screening and prevention after a pregnancy complicated by gestational diabetes
Source: PLoS One. 2022 Nov 18;17(11):e0277330. doi: 10.1371/journal.pone.0277330 (PMC9674174; doi:10.1371/journal.pone.0277330)
Supplement: S1 Appendix — (DOCX) [file pone.0277330.s001.docx]

**S1 Appendix. Interview guides**

**Patient interview guide questions on barriers and facilitators**:

## [For both screened and unscreened groups]: Was there anything that got in the way of your getting tested for high blood sugar, or diabetes, after pregnancy?

Prompt: [For those within one year of delivery] Please think about the 3 months after you gave birth. What were the most important things to you during that time?

Prompt: [For those more than one year after delivery] Please think about time after your baby turned 1 year old. What were the most important things to you during that time?

Prompt: What were some of the things that made it hard for you to follow-up on your health during this time? In what ways did they affect your ability to get checked for diabetes?

Prompt: Which of these things had the biggest effect on you?

Prompt: Of all the things you have to do after giving birth, where does getting tested for diabetes fall?

Prompt: How have your prior experiences with the healthcare system affected your ability to get follow-up for your health?

**[For the screened group]: What types of support made it easier for you to get tested for diabetes after pregnancy?**

Prompt: Please think about the time after you gave birth. What things made it possible for you to get tested for diabetes?

Prompt: How long after you gave birth were you tested for diabetes? What test did you have (if you recall)?

## [For the unscreened group]: What types of support would have made it easier to get tested for diabetes after pregnancy?

Prompt: Please think about the time after you gave birth. What could your family, doctors, nurses, health center or others have done to help you get tested for diabetes after pregnancy?

## If you were health center director, what kinds of things would you do to help women get tested for diabetes after pregnancy?

Prompt: What time of day was your OB visit after you gave birth and could you get tested for diabetes the same day?

Prompt: Do you and your baby see a doctor at the same health center? If not, what affects your choice of care for you and your baby?

Prompt: If anything were possible, where could you go to be tested for diabetes that is easier to get to than your doctor’s office? What are some places you go frequently?

## If your doctor or healthcare team were here, what is the one thing you would tell them that would have made your experience during and after pregnancy better?

**If time allows:**

*Prompt: What supports would make it easier for you to eat healthy?*

*Prompt: What supports would make it easier for you to exercise regularly?*

## Is there anything else that you would like to add that we haven’t covered?

**Provider and Staff interview guide questions on barriers and facilitators**

**Focusing only on what you can control, what are some of the things that make it difficult for you to help your patients get the follow-up they need after pregnancy?**

[Provider] Prompt: The post-partum time period is a time of care transition. Describe some of the ways providers communicate after a pregnancy.

[Provider] Prompt: What do you have in the way of support (staff or electronic) to assist with follow-up of patients after pregnancy?

[Staff] Prompt: Describe your role in helping patients navigate the healthcare system during and after pregnancy.

**What do you perceive to be the biggest barriers for your patients receiving post-partum screening for diabetes?**

Prompt: What concerns do your patients express about the challenges they face beyond their immediate healthcare?

**What system changes would you recommend to help women get tested for diabetes after pregnancy?**

[Provider and Staff] Prompt: What are your current procedures for scheduling post-partum follow-up for women with GDM? What time of day are they scheduled?

[Provider and Staff] Prompt: How are women followed up if they miss a post-partum follow-up visit? Or if they miss a post-partum screening test?

[Provider and Staff] Prompt: Are there settings outside of the clinic that you think would be good places for patients to have the option for screening? What do you see as the biggest challenges to putting this in place?

[Provider] Prompt: When you give a patient a lab slip to have diabetes screening, what follow-up protocols, if any, are available to help the patient get the test done? What protocols would you like to have?

[Provider] Prompt: How is type 2 diabetes screening currently tracked in the

electronic health record? Are there electronic decision supports that you feel would be helpful?

[Staff] Prompt: What supports would you like to have in providing education for patients?

**[Provider] What opportunities are there for collaboration among providers to improve post-partum follow-up?**

Prompt: What processes should be implemented to improve transition of care communication among providers?

**If time, ask**

*Prompt: What do you perceive to be the biggest barriers for your patients in changing lifestyle to prevent diabetes?*

**Is there anything else that you would like to add that we haven’t covered?**
