# Supplementary material for: Barriers and facilitators to diabetes screening and prevention after a pregnancy complicated by gestational diabetes
Source: PLoS One. 2022 Nov 18;17(11):e0277330. doi: 10.1371/journal.pone.0277330 (PMC9674174; doi:10.1371/journal.pone.0277330)
Supplement: S2 Appendix — (DOCX) [file pone.0277330.s002.docx]

**S2 Appendix. Additional Exemplar Quotes**

**Barriers and Facilitators---Society**

| **Barriers** | |
| --- | --- |
| **Sub-theme** | **Participant perspectives** |
| **Insurance issues** | I thought I would still get Medicaid…with me having her, you know...But I don’t understand why I don’t and some people I know…have kids and they still have Medicaid so I don’t understand that (participant 4, screened, delivered > 1 year).  With Medicaid …, they allow you to get care and keep like your full coverage Medicaid for so long and then after for so long, they took me off for a while…So if you can’t get the full health coverage, it is kind of hard to go to your OB-GYN or endocrinologist or something…Because it only covers so much (participant 12, screened, delivered > 1 year).  There was a nice gap maybe like 3 or 4 years where there was no insurance. It was hard. I mean you can’t just go on and get a regular checkup. I was checking myself during that time with the needle that my mother used to ah have for diabetes, that’s how I was kind of like I didn’t have it prior because my numbers were always in the normal range (participant 3, screened, delivered < 1 year).  Now I haven’t seen my my doctor, my regular doctor in years. Like I always go to the Urgent Care if I’m really sick. And, you know, there is no blood work or anything that I have done recently…so health insurance is a thing (participant 50, unscreened, delivered > 1 year).  Sometimes they’re waiting for approval for…Medicaid so they’re almost in limbo and depending when they found out they’re pregnant, maybe they are past that 24-week mark of gestational diabetes there and then they’re waiting for insurance and they may already have diabetes. So there’s some, some issues with one, getting them to the doctor early enough in their pregnancy and getting them insured early enough in their pregnancy to be able to manage their gestational diabetes (participant 30HCP, Registered Dietitian).  Sometimes, you know maybe they missed the 2-week and the 6-week and now they no longer have insurance. So they could still potentially afford to get the test done but maybe the visits and the copay you know it depends on the patient (participant 4HCP, Ob/Gyn, Nurse Practitioner).  For my patients, none of them are documented so they, they don’t even have the 60 days… they lose it [Medicaid] right away (participant 1HCP, Family Practice, Nurse Practitioner).  People lose their insurance and then drop off the grid for a while. Or they only have coverage for like women’s prevention. And it can’t just be that for the visit if they just came for diabetes you have to make up something, I talk to them, yes they like the birth control so put that at the top (participant 11HCP, Ob/Gyn, Physician).  That’s going to be an issue for our patient’s because a lot of times even if it’s not very much, it’s still a copay (participant 19HCP, Internal Medicine, Physician).  We’re good about it during pregnancy but then I think the after the follow-up after that 8 weeks….Gets it’s not so as good. Because we just don’t really don’t see them. Unless it’s with the baby. And we’re not usually focusing on how, have you got your insurance? It’s always about the baby (participant 26HCP, ObGyn, Nurse).  We still have a lot of uninsured adults that technically qualify for Gateway but some of that reluctance is…they are afraid they will lose other state benefits (participant 11HCP, Ob/Gyn, Physician). |
| **Employment Struggles** | I had a doctor note they said I could lift so many pounds…. I worked up to 7 months before they made me resign my position...But that was stressful too during the pregnancy because they also tried to fight me for the unemployment (participant 1, screened, delivered > 1 year).  I had started with a new job and I just didn’t want to risk coming in and out the…Doctor (participant 28, unscreened, delivered < 1 year).  My type of job I can’t go into the bathroom for 30 minutes or an hour and pump his milk for him and I can’t sit at home all day with him either right now. I had to go back to work so that was the best choice for him then, so now he is on formula and it’s a little easier (participant 3, screened, delivered < 1 year).  Because I have multiple jobs…some of the jobs had already you know put their schedule in like a month or so in advance. So… When it [time for follow-up] comes, there is no way (participant 48, unscreened, delivered > 1 year).  I am working two jobs. I have a job at the law firm as a foreclosure specialist and I do grocery shopping for Instacart for my second job… So I am working about 14 hours a day. On the weekends I am working 12 hours, 10:00 to 10:00… then I am going to grab something you know. So it is probably you know not that healthy (participant 32, unscreened, delivered > 1 year).  If they just were off six weeks because they had a baby, they cannot take off more because, you know, the blood sugars are all wonky. So there is and not wanting to take off work (participant 23HCP, Ob/Gyn, Nurse).  I think that’s a big problem just…being able to work through their pregnancies and get all the appointments in (participant 27HCP, Ob/Gyn, Nurse).  I think I read somewhere the other day that 25% of new mothers go back to work within 2 weeks of giving birth, 2 weeks. Their body is still aching. You haven’t slept in 2 weeks. So that’s a huge barrier, especially for low income families. They can’t afford to take off 6 weeks, let alone 12… they are losing an income at that point, so taking off more time to go to their appointment (participant 31HCP, Registered Dietitian). |
| **Housing Instability** | I really didn’t get start getting prenatal care with her until about I was 5 months because I had just moved and it was a lot of chaos going on at that time... I came in going into the shelter and going here and going there… when you’re going through, you know, all of that and then you’re pregnant and then you look, you’re, you’re homeless and you’re, you’re this and you’re that and you barely have money for necessities (participant 10, unscreened, delivered > 1 year).  I was stressed due to my living situations. It was a very unsafe environment…we were never supposed to be in the home. It was not clean. It was not sanitary and I could not move then…So it was a lot of stress (participant 11, unscreened, delivered < 1 year).  Trying to keep those numbers updated in our systems so we know that we’re going to have contact and that, and then hoping that their phone hasn’t been turned off (participant 25HCP, Family Practice, Nurse)  Another problem is, the telephones. Trying to get them reaching the telephone sometimes if the phones have been changed or not working or…You know and people moving around (participant 27HCP, Ob/Gyn, Nurse).  They have a number today. Tomorrow it’s cut off (participant 24HCP, Ob/Gyn, Nurse).  I think our patients have other priorities, you know, I’m still going to get kicked out of my house even if my hemoglobin A1c is still 7…it, doesn’t maybe crossover into one of their greatest worries (participant 19HCP, Internal Medicine, Physician). |
| **Food insecurity** | So I couldn’t really afford and my blood sugar was high because I just got to eat what was available to me. I knew where to go I just couldn’t afford it…. I notice if I like go out there versus like I live in South City, the produce and everything out there just to be a little bit better so usually when I go grocery shopping I like to go to like 2 or 3 different store that are at least 20, 25 minutes away (participant 5, unscreened, delivered > 1 year).  I would suggest that WIC … could have more healthier choice for the juice, honestly [juice is] just sugar and color [laughs] (participant 6, screened, delivered > 1 year).  I wasn’t getting food stamps due to the job that I had you know it was kind of difficult for me to transition … And learning how to still eat healthy…you know vegetables and stuff it’s really expensive (participant 31, screened, delivered > 1 year).  So during the pregnancy, it [WIC] was not helpful because a lot that I couldn’t have. The nutritionist did tell me certain thing, you know, that can help me far as the carbs and counting the carbs. She did do that. That was helpful. But the food they was giving me, it was not helpful during the time of diabetes (participant 37, screened, delivered < 1 year).  Healthy food is a lot expensive and quick meals are just cheap…I would take the junk foods and the fatty foods and meats and stuff like that and just portion it a little bit less than I would normally do when I wasn’t pregnant. Instead of going out and buying all the healthy, healthy food (participant 52, unscreened, delivered < 1 year).  I think that the one of the biggest things of all, it is expensive to eat healthy and, I think yeah I just think that that, that’s part of the problem (participant 6HCP, Ob/Gyn, Nurse Practitioner).  The customizations are, are still pretty limited…WIC provides concentrated juice to moms…that’s extremely high in sugar, so that is something that we could remove completely…we could just reduce the amount that’s offered on the package (participant 29HCP, Registered Dietitian).  As for adjusting like the high sugar content foods, if the juice is chosen to be eliminated, there really isn’t a substitute for that money. So it’s you use it or you’ll lose it (participant 30HCP, Registered Dietitian).  I feel like a lot of our patients live in food deserts where they can’t get access to decent diets (participant 9HCP, Family Practice, Physician).  Not only food but a lot of our neighborhood don’t have grocery stores. They get their food at Family Dollar (participant 11HCP, Ob/Gyn, Physician).  If you are so poor that you can barely afford food, you are going to buy what you can afford and it is not necessarily healthy things (participant 23HCP, Ob/Gyn, Nurse). |
| **Transportation challenges** | The only thing that I wish I had had was my job’s vehicle. Because it was like, once I lost that, that was when I went on the path of going down, off track, and it is hard to get back on track because I am still not on track since the day (participant 1, screened, delivered > 1 year).  I had a problem with transportation. That’s another problem too. It’s a big thing is transportation why people cannot come out, especially with low income (participant 37, screened, delivered < 1 year).  My car was falling apart, everything. And then I got these kids don’t like catching the bus (participant 42, unscreened, delivered < 1 year).  We usually have transportation already arranged for them. It’s not necessarily really convenient. You know they’re going to get picked and they are going to get home hours later, so yes it is there, but it’s not really (participant 13HCP, Ob/Gyn, Physician)  Like if insurance is available with transportation, I think that would help as well. Because a lot of them, it’s early they have to be here. I always try to get them here at 8:30 because they have…To fast. You know so and then if they don’t drive, they have to who’s going to pick me back up 2 hours later….I don’t really think too many other things that are, you know, preventing them from coming into care….the biggest barrier is either them getting here (participant 24HCP, Ob/Gyn, Nurse).  I think transportation can be a barrier too, for the woman to get back in here to see the doctor (participant 28HCP, Registered Dietitian)  When they [Medicaid] arranged private transportation for the patient whether or not they’re going to get here on time for their appointment is kind of like a flick of a coin and then, you know, we only have a 10-minute late period (participant 25HCP, Family Practice, Nurse). |
| **Lack of Childcare** | I set up doctor’s appointments for me but then I don’t end up going because no child care….It has been very hard to get follow-up care… I don’t have…a babysitter …some physicians, you know, they won’t see you with 4 children in the room (participant 10, unscreened, delivered > 1 year)  When you’re trying to take care of yourself like that, you do want some privacy. You don’t want to be like hey get over here. You sit down please. Could you stop (participant 10, unscreened, delivered > 1 year)?  You’re struggling then you got to try, you know, get to appointments and you have to figure out who to watch your kids. I cannot bring out all these kids with me to the doctor. They’re running around in the clinic. They’re talking out loud. They’re going down the street, around the corner and always (participant 37, screened, delivered < 1 year)  I have an almost 5-month-old plus I have a 3-year-old. I can’t get nobody to keep them (participant 44, unscreened, delivered < 1 year).  I just needed that that time [laughs] after I had the baby to make sure okay I have somewhere for him to go because you know most doctor’s appointments don’t allow you to bring a child in with you….You know you have a lot of parents that struggle with childcare. You don’t have that one family member that could come tag along with you and you know make sure…Everything is okay (participant 48, unscreened, delivered > 1 year). |
| **Facilitators** | |
| **Sub-theme** | **Participant perspectives** |
| **Community organizations** | I had the people come into my home and we used to go over what I ate the night before, I used to have to jot down, I had a folder. They also get me a little thing to count myself too, but I had a folder and every time they came, we went over how that food, how that meal made me feel versus certain other meals and they will give me an idea of what I should eat for dinner. (participant 2, screened, delivered > 1 year)  I had a Parents as Teachers that used to come to see me and my daughter…when I had somebody there to help guide me through it, it was easier but, doing by myself, I was more scared (participant 44, unscreened, delivered < 1 year).  I had Nurses For Newborns…. so I feel the people that was in my corner…they were my support system. Helping Hands help. A lot of people don’t know about Helping Hands (participant 11, unscreened, delivered < 1 year).  She just asked me if everything is going good…after the baby is here… I don’t like to feel like I want to be a burden to anyone, you know…I don’t want the attention on me…(participant 48, unscreened > 1 year)  We applied for scholarship at the YMCA…so it is affordable, spent 20 dollars a month for five people in our family….I have also found a cooperative as well. There is a coop closer store called City Market. ..they have vegetables and stuff and they accept PVC and you get more for your dollar….They talk about a lot of that stuff and find solutions to our problems that we have as a whole and that is especially one thing you know that like, you know, supporting Black businesses (participant 31, screened, delivered > 1 year).  They just quit coming because they are like well I don’t have insurance. Because they don’t even realize that we have a sliding fee sometimes (participant 4HCP, Ob/Gyn, Nurse Practitioner).  Nurses for Newborns is about a year…and then Raising STL is a combo of Nurses for Newborns plus Parents for Teachers..So that goes through 7 years of age for the kid…some of our patients don’t want to sign up because it’s such a long time, they don’t want to be followed for that long (participant 11HCP, Ob/Gyn, Physician). |
| **Government programs** | I’m on Medicaid but I’m on Home State. Home State they said when they found out that you’re pregnant, they send out people. They send out nurses and stuff especially when they get a little bit about your history which are medical so not only was my doctor following my health…My insurance company was following my health (participant 11, unscreened, delivered < 1 year).  They just automatically put me on Gateway… that’s what I was on before I was pregnant with her (participant 4, screened, delivered > 1 year).  It was much easier to eat a healthy diet when we were on SNAP (participant 22, unscreened, delivered > 1 year).  They are going to give you a certain amount and make sure it is healthy so WIC has escalated, so it’s good (participant 46, screened, delivered > 1 year).  WIC is a lot easier than Medicaid (participant 24HCP, Ob/Gyn, Nurse).  We have to make sure, you know, she focuses on getting sure everybody …gets on Gateway to Better Health…  Or that we make sure that transition happens for them because of that, the temporary running out at the, at the time so that we can keep them in the system because that’s our goal so we’re focused on that (participant 27HCP, Ob/Gyn, Nurse).  We try to get people into the WIC program within the first trimester because we see better results as far as healthier babies and pregnancy outcomes … with earlier nutrition support and education, but that doesn’t always happen (participant 28HCP, Registered Dietitian).  So WIC does promote foods that are a little more diabetic friendly, fresh fruits and vegetables, beans, peanuts butters are good, iron, a good source protein food source you’d want in a diet, eggs (participant 30HCP, Registered Dietitian). |

**Barriers and Facilitators -- Healthcare system**

| **Barriers** | |
| --- | --- |
| **Sub-theme** | **Participant perspectives** |
| **Care fragmentation** | I can do the wait for me. Kids don’t really have time for that, so that is why I don’t stand them to where I go (patient 46, screened, delivered > 1 year).  They didn’t test me until after she turned 1…..The doctor was pregnant too and she went on leave….And I guess the new doctors didn’t check the charts until now (participant 13, unscreened, delivered < 1 year).  A lot of times for me it is they are spending that first year bringing their kids to all of their kids appointments with me. And then finally I am like it is probably time for to come in for yourself. Right? So, it’s very rare that I’m, that I see very many people like within even the first 3 to 6 months postpartum unless they have something very specific…if I don’t have a reason to enter your chart, I may or may not remember that you had gestational diabetes without looking at your problem list and seeing it there (Participant 8HCP, Family Practice, Physician).  I think that’s the hardest part about OB is like once the baby is out, okay you shift back to your GYN mode instead of your OB mode (Participant 16HCP, Ob/Gyn, Physician).  If they did follow up somewhere else they probably don’t have records just like if they deliver at [location redacted] we don’t have records (participant 4HCP, Ob/Gyn, Nurse Practitioner).  Sometimes it is because they want birth control that we get them back in. So like if it is a long-term device…They won’t keep it in the pharmacy for so long so then they’ll call us and say hey we’re going to send it back if the patient doesn’t get it and that kind of triggers like by the way she never called back. That might trigger the process but…there’s not a very good tracking postpartum when they don’t show (participant 4HCP, Ob/Gyn, Nurse Practitioner).  We try to ask them those questions like was it a complicated pregnancy did you have any issues, was there gestational diabetes, did you have hypertension or did you have preeclampsia all that stuff, but a lot of times when they come in, you’re forgetting some of those questions too because their blood pressure is 200/100 now (participant 18HCP, Internal Medicine, Physician Assistant).  You would refer them to the nutritionist and part of the problem I think was that meant another appointment, right? So the patient had to come back to the clinic at another time and instead of just being able to hand them, hand them off when the patient was here (participant 19HCP, Internal Medicine, Physician).  So our EHR [electronic health record] one thing is very annoying about it is the OB problem list does not automatically populate back into say a GYN problem list (participant 11HCP, Ob/Gyn Physician).  I think we find in a community health center that people move from community health center to community health [center]. You know like oh I got my ultrasound at [one center] and then I was at [a different center] (participant 12HCP, Ob/Gyn, Physician). |
| **Scheduling policies** | I think…wherever they have delivered does tell them to follow up with the provider, but again that’s back on their hands to schedule that appointment (participant 4HCP, Ob/Gyn, Nurse Practitioner).  Most patients have appointments for their babies and themselves when they leave the hospital after delivery but if they miss that appointment, accessing the line, and getting an appointment can be tricky (participant 1HCP, Family Practice, Nurse Practitioner).  What’s harder to catch is if they are doing the right thing and they cancel their appointments. When they call to cancel, they don’t always reschedule and if they call to cancel, there is no prompter to us to even be aware that they canceled (participant 8HCP, Family Practice, Physician).  The safety net that we currently have is when somebody no shows our MA is supposed to send us a task so that we then can make a decision looking through the chart, do we want them called back. So it’s kind of an imperfect safety net (participant 9HCP, Family Practice, Physician).  We call them and try to reschedule them. If they don’t answer it, we send them a letter…Ah so typically they get about 2 letters and 3 phone calls. If no response after that, we just stop trying (participant 24HCP, Ob/Gyn, Nurse).  Once they’re outside of that postpartum window or whatever … the patient really has to make an effort in order to maintain the follow-up that they need (participant 25HCP, Family Practice, Nurse).  It needs to be 2 hours, so it’s something…that needs to get scheduled (participant 26HCP, Ob/Gyn, Nurse). |
| **Appointment time constraints** | I know the first time I wasn’t really able to get a lot of support and help there… because the doctors there seem like they had too many other…People to focus on that so I just felt more, you know, not safe per se (participant 25, screened, delivered > 1 year).  So back when I was working with [clinic name redacted], a lot of time I used to let it, let my appointments slide because…you’re literally two hours or later after your appointment waiting, so you’re like no I don’t want to go to this clinic (participant 36, unscreened, delivered < 1 year).  One thing that is difficult is sometimes things do take a very long time. I felt like maybe, you know, having appointments that are too far apart or (sighs) test results take a long time … they’re just extremely like busy and…And sometimes under staffed so sometimes when it comes to appointments that you want to find out something relatively quickly, you have to wait on (participant 53, screened, delivered < 1 year).  Because you end up seeing them every week, so then I kind of leave some of the more overwhelming information like…oh what are the risks to the pregnancy and I kind of tell them the basics…I am going to like spread it out a little bit partly because I don’t have time at their first visit (participant 14HCP, OB/Gyn, Physician).  I think community health workers would be nice…Because they actually can go to the house (participant 24HCP, Ob/Gyn, Nurse).  Well so it is usually like we are pulled over to the exam room and that woman does not have a lot of time you know because usually they are hungry…and they have been here for hours already (participant 28HCP, Registered Dietitian).  Sometimes it feels like you’re in the room for much longer than 15 of the allotted minutes…. there is so many little things that you cannot do in the 15 minutes (participant 18HCP, Internal Medicine, Physician Assistant).  If they don’t speak English, it takes longer. If they’re having trouble, I mean it’s so hard to say. I would say at least 30 to 40 minutes each visit (participant 24HCP, Ob/Gyn, Nurse).  It’s hard with the time with constraints, too. When you’re trying to get everything in there (participant 27HCP, Ob/Gyn, Nurse).  In the limited time that we have because we really only have about 10 to 15 minutes with them at their subsequent…visits (participant 31HCP, Registered Dietitian). |
| **Staffing limitations/needs** | We don’t have a nutritionist specifically at our facility… we just have the nurse who does the nurse education (participant 4HCP, Ob/Gyn, Nurse Practitioner).  I think it would be nice too [if] all of our nurses had some type of baseline training in nutrition and diabetes (participant 11HCP, Ob/Gyn, Physician).  I have had the privilege of working at 2 different sites with 2 nurses with very different skill sets and my nurse at my main site understands diabetes and is able to counsel the patients very well, and teaches them about their diet, how to check their sugars, how to modify their sugars based on what they eat, and the other one has, has no skill sets so even just having them all have kind of a standard education, in kind of how we manage diabetes in pregnancy (participant 16HCP, Ob/Gyn, Physician).  It would be nice to have a nutritionist… At my last practice… we had cross trained staffs as a Behavioral Health consultant or a couple of the nurses were cross trained as diabetic educators…So you take all the courses and got certifications (participant 18HCP, Internal Medicine, Physician Assistant).  I think home visits would be awesome. If there was some sort of diabetic educator that traveled (participant 23HCP, Ob/Gyn, Nurse). |
| **Facilitators** | |
| **Sub-theme** | **Participant perspectives** |
| **Care coordination** | Oh well no, they called me yeah, the night prior like it’s a fasting test. I got there at 8 a.m. yeah. That wasn’t a challenge (participant 3, screened, delivered < 1 year).  I actually put it on hold and you know order it or something like that just so in case they don’t show up at their 2 week visit. And there are usually notes in the chart of what the plan is, but I think it’s a probably a shared responsibility whoever sees her knows that that is what the plan is (participant 4HCP,Ob/Gyn, Nurse Practitioner).  We do huddling in the morning stating the goal for each patient with our medical assistant and so if I know oh this person didn’t show up but this is what they were supposed to be in for and we need them back again, then I’ll call the patient, I’ll have my nurse call the patient we’ll, I’ll have the scheduler call the patient to try to get her back in. Because then I try to make it a personal point to like some have said, figure out like what is the barrier (participant 2HCP, Family Practice, Nurse Practitioner).  I think the nurses do a pretty good job of calling every patient that delivers and getting their postpartum [appointment] scheduled (participant 6HCP, Ob/Gyn, Nurse Practitioner).  I will try to tell them…if you leave the hospital you know if you don’t deliver at [location redacted] or if you leave [location redacted] without a 2-week appointment just call us…but then if they do come in for their 2-week postpartum and then I’m like hey we’re going to give this 2 hour diabetes test, then I will make a point to like try to schedule it first thing in the morning (participant 3HCP, Ob/Gyn, Nurse Practitioner).  Well, the great thing about electronic is that we all have access to each other’s charts like instantaneous…it’s easy to get messages to other providers too (participant 4HCP, Ob/Gyn, Nurse Practitioner).  Warm hand offs… I will have my medical assistant walk them over there and get them an appointment (participant 21HCP, Internal Medicine, Physician Assistant).  We try to kind of cluster the visits in the beginning because that’s where you’re really trying to learn…And understand and then once they…feel more confident in checking their blood sugars and counting the carbs…then we just kind of follow up and we usually do more just like less intense and more just same day pop in. So it’s coordinated care…they can just ask the dietitian some questions while they’re here with the doctor (participant 28HCP, Registered Dietitian).  We have community health workers now …they’re on board to do those home assessments with the nurses so that can provide more education potentially teaching of using a glucometer or thinking about what the resources of food in your area or exercise or you know so they are just kind of a MA on the streets so to speak (unidentified participant, focus group 2).  I have a very good relationship with our Internal Medicine Department at my same site. And I tell patients so I’m more than happy to take care of you during pregnancy and we’ll be happy to do your gynecologic care, but I also think you should have an internist (participant 12HCP, Ob/Gyn, Physician).  I typically try to schedule the postpartums with a Family Medicine doctor. Because in a way they can do their postpartum, do their 2-hour and they can continue to follow them up after that. You know, and if I know this patient had gestational diabetes, I will try to get them hooked up with the Family Medicine doctor so the baby can see them for peds. So that everything can be done at one time (participant 24HCP, Ob/Gyn, Nurse).  We may give the pediatrician a heads up. When this baby comes in, we really need to see mom. We’ll put a note in the baby’s appointment (participant 26HCP, Ob/Gyn, Nurse).  I think it’s something that with my experience here with the low income population, if they’re kind of used to having those home visiting nurses come in those first few weeks with their baby…Those visits tend to focus on the baby… I think that’s a model that could be incorporated easily for them like if you had a community health worker or a home visiting nurse checking with mom and have her read her blood sugar logs or get a brief diet history even if it was brought back to a dietitian who could better assess it (participant 31HCP, Registered Dietitian). |
| **Pregnancy support groups** | With my first pregnancy they had classes…you meet people in there and I think that they should do the same for the people that going through the same thing, they may not have family or have anybody you can talk to that person and… People can get close to other people and you know I think it would be good to have something like that (participant 43, screened, delivered < 1 year).  All the pregnant women. It was like a little round table…. it made it easy because you see your doctor, you get your WIC, you get your blood pressure checked, you work out with the other moms and dads (participant 46, screened, delivered > 1 year).  You know, this was my first time but they done had it before or they all already had, you know, diabetes. So hearing they outtake was, you know, helping me (participant 44, unscreened, delivered < 1 year).  They send me to a class ah which I still owe for because I didn’t have insurance back there… it was like $800 class….probably still get the bills for it (laughs) (participant 50, unscreened, delivered > 1 year). |
| **Patient education materials** | Like one booklet of stuff that’s all going to be helpful….Instead of a bunch of papers… instead of a ton of different things that they’ve collected (participant 24, unscreened, delivered > 1 year).  On the American Diabetes Association website… I sometimes will give to patients I know do have internet either on their phone or at home where they can calculate what their weight should be and it’ll help them to kind of carbohydrate track … a lot of the patients I don’t feel are candidates for that. I’m just trying to give them like little pieces of low hanging fruit to be able to kind of improve them slowly but surely and kind of wade them into the deep end (participant 21HCP, Internal Medicine, Physician Assistant).  I even printed out like Indian diet. So you know because I know they eat different foods. I just, you know, kept looking online and just finding different resources and kind of created that….I have ordered stuff from CDC that is all free and just stuff from the internet (participant 23HCP, Ob/Gyn, Nurse).  We do have [EHR] handouts … that are very good but then that depends upon the patient to read it… And that’s where I think that may not be happening (participant 20HCP, Family Practice, Physician Assistant).  We talk about what they have been eating and what is a carb and we go through label reading and kind of more portions and like using the plate and so that is where it becomes very more tailored to that person as far as how … how the person even is comfortable with reading or their education or if you are just using more visuals…you know a fist or like kind of proportions rather than trying to count carbs (participant 28HCP, Registered Dietitian).  We have found some good picture resources and that were in other languages... We also use food models here too. Which that can be powerful but it doesn’t always, I find that it doesn’t generalize well (participant 31HCP, Registered Dietitian).  I would love to see the WIC program come up with … simple education resources for helping a mom with gestational diabetes know why it’s important to monitor it and control it and like her risks of type 2 diabetes or later her baby’s risk and basic simple things she can do in her diet in case we are the only care provider. Because if a mom doesn’t have the resources to see a dietitian outside of WIC, we’re her only one and the WIC program like I said we’re, we’re all dietitians here so we can provide nutrition therapy but not all WIC agencies have an RD in that role (participant 31HCP, Registered Dietitian). |

**Barriers and Facilitators --Interpersonal**

| **Barriers** | |
| --- | --- |
| **Sub-theme** | **Participant perspectives** |
| **Negative experiences with HCP** | I had a few bad experiences, yeah. Uh huh like just people being rude, though. Not really too, you know, too much but it is, people just being rude (participant 10, unscreened, delivered > 1 year).  They didn’t listen... I know my body, it’s my body…You just work on my body. … So I didn’t, I didn’t know a lot that was going on because they never, they never talked to me about my health issues, okay? So I’m back in the diabetic situation again when it could have been prevented. So if your healthcare providers don’t talk to you about diabetes or about things you can do or tell you all the information, then you, you limbo…Because we bank on the healthcare providers to tell us. We don’t know. I can research so much but then what if I don’t research something you told me then I did miss the whole chapter of something that I needed to have (participant 11, unscreened, delivered < 1 year).  I continued telling her something was wrong. But I was brushed aside because I also have bipolar disorder …they did do an ultrasound to check if something was wrong and he had a placental abruption … but I was not taken seriously. I have lost a lot of trust in that OB-GYN (participant 22, unscreened, delivered > 1 year).  I can’t remember because it was so long ago but I do remember at that appointment, my mother-in-law was there and my husband was there and something happened with the doctors that just made us uncomfortable when she came in, when she talked and like I say, I honestly can’t remember but when we all left, we all said the exact same thing… I know doctors are here to do their job and, you know, thank God for doctors but it’s not always consistent with every single doctor (participant 34, unscreened, delivered < 1 year).  I think in general a lot of the nurses and doctors don’t really listen to their patients which is really that’s very, very important like with my first pregnancy. I’m like I’m almost certain  I had diabetes and I don’t know if that was the reason why I actually ah I almost bled out. I think I lost like 3 or 4 liters of blood when I had a C-section and I had to get a blood transfusion and I figure, I think all of that could have been prevented if I, if the doctor would have just listened to me... Because I was telling him you know I think I am gaining too much weight and I was passing out all the time and he kind of just blew it off….. when I took the class I was instructed like if your blood sugars goes above this number, you need to call us or go to the emergency or you need to come in like I can count, there was at least 5 or 6 times from the time that I was diagnosed to when I delivered where it was about that number they gave me and they kind of just told me to go lay down and drink some water…. I think even after like the first experience with that doctor and then like the second time just did not really trust doctors and not with having my children (participant 5, unscreened, delivered > 1 year).  They tried to kill me, I believe (laughs) with this infection….I was actually swelling at the hospital. I could, my legs were this big at the hospital. They did an ultrasound on me. They told me nothing wrong with me. And sent me home. … I kept telling them this is not normal…And I’m not a doctor. I’m not, I didn’t go to school for no doctor. I’m like this is not right. I’m, I know this is not human….. It was just a whole bad experience all the way around, boy. they didn’t listen to nothing I’d tell them (participant 37, screened, delivered < 1 year).  They are not telling me the results and then when I got in there, the nurses said okay did the doctor tell you what he found on your test results. I’m like no, nobody has told me anything …when I got into the maternal and fetal ward, they broke it down, letting me know that gestational diabetes is basically your sugar is going high … so they was basically like this is what you have to do. They said did your doctor explain it to you? I’m like doctor didn’t say nothing to me. He just told me I had to come over here and talk to you all (participant 29, screened, delivered > 1 year). |
| **Cultural differences** | One of the things patients tell me or we talk about is I think food is a big barrier so culturally first of all like the people that I have the hardest trouble with, with blood sugars are some of our patients who have a rice-based diet like a lot of our patients from Burma and Nepal like you can say not rice and that, that is what they eat every single meal (participant 12HCP, Ob/Gyn, Physician).  I mean they eat so badly. I mean they eat rice, noodles, and bread and bread and more bread, and I mean they’re all at very high risk (participant 7HCP, Family Practice, Nurse Practitioner).  Nobody thinks that these like very big children are a problem either... especially Hispanic folks are like…big baby is a good thing… You know for *gordito* (participant 12HCP, Ob/Gyn, Physician).  It’s…a lot of different cultural that we have here.  They often base how well they are doing on their symptoms, and it’s hard to explain to them that when you have symptoms it is super bad… I think that re-education is key (participant 10HCP, Family Practice, Physician).  There is you know always barriers of like and I know that God wants you to not eat or whatever but he maybe really wants you to eat (participant 23HCP, Ob/Gyn, Nurse).  For example, a lot of the men and women I see here are Black…And they a lot of them that I talk to actually are concerned with gaining weight…I want to be bigger. I have heard that a couple of times or losing weight is associated I think with some patients mind with some devastating disease like cancer or tuberculosis so they get spooked out by that…(participant 19HCP, Internal Medicine, Physician)  Because my other 5 pregnancies were fine so I know what, you know, why all of a sudden I come, you know, to America and now  have this… You know, diagnosis… So I think sometimes it’s just like, you know, culturally it just doesn’t really make sense to them… because they already, a lot of, you know, people from other countries feel like we over test things anyway (participant 24HCP, Ob/Gyn, Nurse).  My Vietnamese [patients] could be 100 pounds but they eat rice and noodles, you know, so all these are high carb foods. So for them this is just a way of life … trying to get them to understand like…you can eat a smaller portion of rice…and more vegetables… You know, just try to make it fit into their culture so it won’t make them feel weird (participant 24HCP, Ob/Gyn, Nurse).  I see the different cultures directly affect how a patient reacts to their diagnosis and what actions they take (participant 25HCP, Family Practice, Nurse).  They go to international food stores so then I’m taking it a little bit of a step further and maybe no one’s really covered that in their primary care visit because it’s usually like here’s the recommendations, but it isn’t followed up by tailoring for them so that’s where like I feel like the dietitian is important in healthcare because they can really ah make it full circle for them and make it work better for their health management (participant 30HCP, Registered Dietitian). |
| **Communication challenges** | The biggest challenge I have is communication because almost all of my patients are non-English speaking (participant 7HCP, Family Practice, Nurse Practitioner).  We have people who can’t write so I tell them to draw pictures, take pictures, show me what you’re eating (participant 24HCP, Ob/Gyn, Nurse).  I had a patient not too long ago who she, I think the big barrier was she didn’t speak English, she was Nepali and, she did not read or write either. But there was also like she didn’t really understand like what a snack was, like water was not a snack like what is a meal and there was like a whole like other like component of…nutritional counseling (participant 3HCP, OB/Gyn, Nurse Practitioner).  My practice specifically most of those patients don’t speak English. So that’s all done through a translator. Which just seems, I mean I know it is a ton of work for my nurse. So I can just imagine how overwhelming it is for our patients who often too have low literacy (participant 12HCP, Ob/Gyn, Physician).  I have a lot of different nationalities so a lot of Nepali, Burmese, they don’t, you know, speak English. It is really hard to find non-English education…some people just cannot like read all of that. I mean some people cannot read. So you know especially I have the English and Spanish everything. But with the other languages like Arabic and Nepali, Burmese, they are really hard to find anything (participant 23HCP, Ob/Gyn, Nurse).  I feel like language barrier can be an issue at times because you can’t necessarily follow up with that person by phone because you don’t speak that language so what you get to that person is in that visit when you physically can’t get them in…the room with you (participant 30HCP, Registered Dietitian).  I think we counted 29 different languages that we have here, so it’s virtually impossible to get every material in every language so a lot of it, you know, if you have a non-English speaking patient with gestational diabetes, a lot of its verbal through a translator…And it still can get lost in translation plus it’s very, very time consuming (participant 31HCP, Registered Dietitian). |
| **Lack of family or friend support** | Just not have ah everything piled on me [laughs].Because I’m only one person I can’t, you know, but it seems like my arm is here, my arm is there, my leg is over here, and then my leg is over there. My head is like this and [laughs].Yeah, I only have one sister and one friend like my best friend that I call my sister (participant 10, unscreened, delivered > 1 year).  I was in a really bad marriage at the time so I didn’t really have a support system (participant 5, unscreened, delivered > 1 year).  When I had it, I was the only person out of my family and friends who had pregnant diabetes… everybody thought I had just diabetes and I was like no…It is pregnant diabetes. I might have it after and I might not (participant 46, screened, delivered > 1 year).  He [husband] didn’t go on diet with me [laughs] which is something he expects when he’s trying to lose weight so I feel kind of mad about that [laughs] (participant 22, unscreened, delivered > 1 year).  People be getting mad at me because I want go to the gym and work out every day. It’s like why do you always have to go? What you going there for? You don’t need to go (participant 4, screened delivered > 1 year).  My doctors told me that too because I was having challenges at home with kids and my husband had played a part in my levels being the way they were too… I was stressed. I was very unhappy. My husband done things to me…that pregnancy was way worse than any, my other three (participant 11, unscreened, delivered < 1 year).  Because that’s a lot of people, they don’t have people who will wait. Like some of them live far…You know. There’s nobody wants to wait on them 2 hours, you know (participant 24HCP, Ob/Gyn, Nurse). |
| **Competing priorities** | I go to work from 6:00 to 3:00 then after that I feel like I need to be at home with my baby or whatever and, yeah that is about the only challenge that I really felt like that I had, uh me not, not being able to get any sleep (participant 1, screened, delivered > 1 year).  I wanted to put him first even though I know my health is more important to make sure I’m healthy for him but he’s more important to take care of. I’ll just sum it up, I have no time (laughs). And I’m always tired (participant 52, unscreened, delivered < 1 year).  It’s hard to keep up with the diet. His health is always more important to me than my health (participant 24, unscreened, delivered > 1 year).  It [diabetes screening] just kind of actually got pushed on a back burner .Because I didn’t really think much of it after that. After life was just so hectic at the time. So I mean that was like the last thing on my, it was still in the back of my head but it wasn’t at the part with having a newborn (participant 25, screened, delivered > 1 year).  It was like they was just overwhelming then I am like I am trying to figure out how to get my daughter of school and I am still at the doctor’s office. It was like it is just so much all in one day (participant 29, screened, delivered > 1 year).  Dealing with the twins at one time and no, lack of sleep [laughs] I drink coffee all day long, that’s why I’m antsy, because I don’t get any sleep. I haven’t had sleep for the last three months. I have one when one goes to sleep then the other wakes up, I’ll make bottles all day and change diapers and I come home my kids come home from school. We do homework and then they play with the twins also, and then they rotate the twins also. Then they screaming and hollering, my twins, doing this, and one of them is crying (participant 37, screened, delivered < 1 year).  You know with doctor’s visit for the baby, appointments for the kids, going running to the school. I mean that was like nonexistent for me to just be like oh you got to go and do this test or whatever (participant 48, unscreened, delivered > 1 year).  So if for some reason like we have to eat out like once a week for fun…. But more than twice a week, I feel terrible. I feel guilty...I’m failing my family [laughs] … I know I was failing them… From working so much (participant 50, unscreened, delivered > 1 year).  That’s really my priority, like my first, it hasn’t really been other things that should be higher on the list but I’ve just basically been trying to make sure that my kids are okay (participant 53, screened, delivered < 1 year).  We have patients who have just absolute chaos in their lives. They’re struggling with depression. They are in situations with unstable housing…when they come in…the visit is dealing with their depression, hooking them up with behavioral health consultants,…dealing with a child who is having behavioral issues at school…that stuff makes it hard to keep on task of things that are more preventative (participant 9HCP, Family Practice, Physician).  But if you have a choice between buying food for your children or buying this glucometer…You know, you know what choice you are going to make (participant 23 HCP, Ob/Gyn, Nurse).  They get caught up with life in general until you and their next issue arises and you are like oh great, 6 months ago you were supposed to come back (participant 2HCP, Family Practice, Nurse Practitioner).  Again, it’s just so hard. I mean they deal with so many challenges which are, they’re overwhelming. I mean they just are and if you know they just have to put into their own minds exactly what they can handle for that day and sometimes this, this is not, this is not it (participant 6HCP, Ob/Gyn, Nurse Practitioner).  For those patients it’s usually like a snowball effect. It’s not just like 1 thing in their life that they can focus on. It’s like 10 things like they’re being evicted and their car is broken down and their…You know, trying to start a new job and they just found out they were pregnant and they’re trying to get out of this really. You know, it’s like their life is crushing down on them so like the 1 little thing that we can do to help them and if there were, if there was availability for them to get what they need to…So that, that the diabetes is one last thing that they have to be concerned about (participant 25HCP, Family Practice, Nurse).  Just the patient inertia of, my baby is born. I’m taking care of my baby. I’m taking care of my small kids. Their own follow up becomes much more secondary (participant 8HCP, Family Practice, Physician).  It’s like well now I have my baby and so it’s like I don’t have to take care of myself anymore, you know, and they make it to the infant appointments (participant 28HCP, Registered Dietitian).  Everyone is a transition so balancing those frequent early infant appointments with mom’s appointments. So often mom is not seen until 6 weeks postpartum but then who know, you know, her life’s been chaos. Because of new baby at home at that time, so in general I think there should be more contact with new families with a new baby (participant 31HCP, Registered Dietitian). |
| **Facilitators** | |
| **Sub-theme** | **Participant perspectives** |
| **Positive experiences with HCP** | So everything that he did, he just did it as let’s prevent things. I care about you, I care about your mental state, care about you and your child and he just was there and he saw me sometimes twice a week (participant 12, screened, delivered > 1 year).  She makes you feel like she really cares. She is part of the family. She asks about your kids. She knows your kids by names, delivery. She checked on the kids when I had them…Anything she tells me like make sure you get this tested or make sure your blood test and she stay on me about that (participant 46, screened, delivered > 1 year).  A lot of our family members on both sides, they have mental illness and … I don’t know if it’s like in just like the Black communities but they figure out mental illness is like no it’s just all in your mind…it’s not like it. Like especially after giving birth, you go through a lot of feelings like…but when you try to work past them, sometimes you can’t work past them. This was my first time reaching out to my doctor about it…And it was it was so helpful. He understood what I was talking about as far as getting my health back (participant 36, unscreened, delivered < 1 year).  You know, I had been pregnant the whole time, they nurtured me and all the nurses know me so they wanted to see how the twins were…(participant 37, screened, delivered < 1 year)  Even though it was so many people who would see me, they all knew exactly what was going on. Like I was seeing them the whole time even though I wasn’t (participant 28, unscreened, delivered < 1 year).  They made you feel really special as if you were the only one pregnant…so just making sure that everyone is comfortable and, you know, approachable so that they know I can come over to you and ask you a question, not feel embarrassed about it (participant 25, screened, delivered > 1 year).  That actually worked with me personally, great dietitian…even though I didn’t want to do things she wanted me to do [laughs] but… she sat there and she showed me (participant 37, screened, delivered < 1 year).  She my same doctor for all my pregnancies. I love her. That she made sure she do her job with me. She makes sure, like she act like my mama a little bit. [Laughs] She be on my case (participant 42, unscreened, delivered < 1 year).  Hospitality…The staff was superb…very attentive, made you feel home like you are at home (participant 48, unscreened, delivered > 1 year). |
| **Family or friend support** | My mother literally was counting everything (laughs) for me so I had like a certain amount of carbs, certain amount of vegetables, dessert that was still limited, a certain amount of juice, certain amount of milk, like I never knew how much sugar milk had in it (participant 12, screened, delivered > 1 year).  I had immense support from my mom, she came to stay with me after I lost my job to help me…financially (participant 1, screened, delivered > 1 year).  Got his checkup done and then I started, yeah, they got his checkup done and then his dad, my fiancé came and picked him up…And I stayed there alone and did my fasting test (participant 3, screened, delivered < 1 year).  And then my friend, she’s more like we walk together. We go grocery shopping together. We hang together. Ah we research a lot of ah food things together and stuff like that and she plans my meals for me a lot of the time (participant 11, unscreened, delivered < 1 year).  Yeah, I mean you just need people around that are positive. They understand what you’re going through and that support you in the way like that’s your friend (participant 53, screened, delivered < 1 year).  I like people’s opinions but when there’s so many…Because everybody wants to help you because they see how hard that would be. I think it just gets overwhelming (participant 24, unscreened, delivered > 1 year).  So that’s what basically kept me like doing right because my family was around to help me, be able to remind me to eat right (participant 35, screened, delivered > 1 year). |

**Barriers and Facilitators --Individual**

| **Barriers** | |
| --- | --- |
| **Sub-theme** | **Participant perspectives** |
| **Infant health and delivery complications** | For her to be still in the hospital and have to rush, rush here, rush there, so yeah. It was, it was a stressful time at that time (participant 10, unscreened, delivered > 1 year).  I was trying to breastfeed him but I guess I was not making enough milk as I was supposed to be making and he just started to shake and I was just crying. They were saying that his sugar was low and by me not having enough milk to you now raise his sugar. So I was just stressed…not really focused on myself, you know just…Making sure my child was okay. And making sure he had enough milk (participant 32, unscreened, delivered > 1 year).  I’m so feared for her to get sick…I missed a couple of appointments already...Having her, I don’t want to have her out every day (participant 54, screened, delivered < 1 year).  His readings wasn’t even high enough to even be read by the machine. So you know he cannot help himself and when you see that you caused a child or something that that cannot themselves more, they have no control, they was banking on you to do everything, yeah I am not. Yeah, that was it right there. He had no clue what was going on, none. He could not do nothing for himself (participant 11, unscreened, delivered < 1 year).  Her health [was most important]. Make sure she was alright. I found out she was born with the same thing, the heart murmur. So now I got to keep her going back to her for appointments because hers hasn’t closed up yet too and they’re trying to see why it’s taking so long to close up (participant 13, unscreened, delivered < 1 year).  Just making sure, that, you know, my baby was healthy because, you know, she was sick, so and then just keeping the girls safe (participant 31, screened, delivered > 1 year).  So then the C-section had me depressed… When you get home you know and you cannot walk. You are not supposed to go up the steps…so it was hard because of the pain (participant 43, screened, delivered < 1 year). |
| **Maternal comorbidities and symptoms** | Some of the exercises, it was kind of hard to me because I had a lot of back pain with my pregnancy…so I did more walking and squatting (participant 43, screened, delivered < 1 year). |
| **Negative Emotions** | It was just overwhelming because ah they would put on medicine and then they would take me off and then they would put me on again and then so yeah, it was just depressing and then it was scary because I didn’t know how it would change my life or how it would change me physically or would they, you know, would it make me sick or would I go to through, would I have prolonged diabetes or it was just a lot (participant 10, unscreened, delivered > 1 year).  I thought that I was okay…I do not know why I did not believe the machine, it’s a machine. I do not know. I just thought I was okay but when I saw that I was not okay, I said I was really upset with myself (participant 11, unscreened, delivered < 1 year).  It was really challenging but once I got into the mix of how to take my medicine and you know just understood how serious it was to not eating certain things or whatever. It kind of got, you know, manageable or whatever but, like I said, it still was kind of scary to deal with (participant 2, screened, delivered > 1 year).  I was supposed to get a checkup in September but I accidently missed it, or I was too afraid to go (participant 25, screened, delivered > 1 year).  Nothing really made it hard for me to follow up on it. It’s just me being me, nervous of the results. You know, there’s a chance of you having diabetes after your pregnancy. It’s so scary (participant 44, unscreened, delivered < 1 year).  It is terrifying to think that thing it can be there the rest of your life (participant 37, screened, delivered < 1 year).  I really didn’t know a lot about it at the time my doctor educated me pretty well but it was very difficult to manage and the whole experience sucked (participant 5, unscreened, delivered > 1 year).  It was draining enough. It’s like okay because you’re constantly like what, what am I going to eat and oh my God what’s in that… It’s like you feel guilty when, when things go wrong on your pregnancy period, you feel guilty (participant 53, screened, delivered < 1 year). |
| **Inaction with regard to guideline adherence** | It’s kind of like, if you felt good and somebody told you, you were sick or take this test so I could just see if you’re sick, would you rather take the test or would you just rather feel good?...that’s the dilemma (participant 28, unscreened, delivered < 1 year). |
| **Unhealthy food and exercise patterns** | I don’t like vegetables. I’ve never been a vegetable, but now I can kind of tolerate some, like the broccoli, but it got to have like cheese in it… or whatever and, but like, at first, it was like I was hearing what they were saying but, like I said I am a picky eater…So, even how much how you tell me you should eat this and that and you cook it and you will be wasting your time because I am not going to eat it because I do not eat it (participant 1, screened, delivered > 1 year).  I always been a healthy eater but it was those cravings that made it hard to do. You crave sweets, so I crave candy (participant 10, unscreened, delivered > 1 year).  When I started eating yogurt, I did not, I could not portion it out so I would just eat the whole container because I did not want to waste it. So that didn’t help. Because I did not waste and it was money wasted (participant 11, unscreened, delivered < 1 year).  When you’re pregnant, you kind of want to eat what you want eat (laughs) (participant 12, screened, delivered > 1 year).  I love bread. You know bread is like the devil (participant 31, screened, delivered > 1 year).  I was like addicted to sodas when I was pregnant…I love water… But…when I was pregnant, I, I really couldn’t drink water, it’s like all I could taste was soda and I guess it was because of the diabetes of why I wanted the sweet taste so much (participant 1, screened delivered > 1 year).  I do not feel like cooking because I’ve been up since 4:15 so I do not feel like cooking, and I’m not going to lie. I will snack something fast to eat for us or something because I literally do not feel like cooking, and I know that is bad (participant 7, unscreened, delivered > 1 year).  The cravings were so strong. It was like I needed my sugars and stuff or I would literally be like angry if didn’t have sugar in my body (participant 35, screened, delivered > 1 year).  My thing is the amounts, that’s what gets me…then I’m like forget it, let me just go back to what I know, and then I’m back to eating the bad foods. So for me, well I adjusted my salads with different colors and stuff. That’s how I try to…Stay healthy because I can’t cook a lot of foods. I have a cookbook and don’t use it (participant 36, unscreened, delivered < 1 year).  It’s hard. Especially when you like other things that are bad for you even though they do taste great [laughs] (participant 4, screened, delivered > 1 year).  When I go to the grocery store, I see nothing but snacks (participant 42, unscreened, delivered < 1 year).  I could eat it for breakfast, lunch, for dinner, for snack. I eat rice. Any occasion. So it was really hard for me because I had to eat these small portion (participant 50, unscreened, delivered > 1 year).  Bring you a whole different menu. Now had nothing good on there. And the food just is blah. Ah I, I don’t know you if you can get better food with the diabetes, whatever (participant 8, unscreened, delivered > 1 year).  We walked around our block and stuff. And I hated it (participant 24, unscreened, delivered > 1 year).  There isn’t anything anybody would do to make me exercise (participant 35, screened, delivered > 1 year).  It’s just motivation …Getting in the habit of getting out and walking and maybe the weather has been excuse [laughs].But I hate summer too.[Laughs] I don’t like to be sweaty (participant 50, unscreened, delivered > 1 year). |
| **Facilitators** | |
| **Sub-theme** | **Participant perspectives** |
| **Children’s wellbeing** | I was doing it for my son so any time you do something for your child, it kind of gives you a little boost and motivation to really want to do it (participant 12, screened, delivered > 1 year).  Once you are pregnant you have a baby you are not thinking about yourself. If you got to sit here and do something to make sure your baby is okay, you are going to do it (participant 46, screened, delivered > 1 year).  Because I have children….And I don’t want them to be obese. I don’t want them to have the diabetes….So if I can do better, I’m going to do better with the food, the portions,… it’s my time to take care of me because I have to be here for them (participant 30, unscreened, delivered < 1 year).  So it was just exciting time for me after she turned 1 to see her develop the way that she has. It’s, it’s amazing to me (participant 10, unscreened, delivered > 1 year).  I’m also going to make sure that they’re [my children] eating right because... I don’t want them to have these kinds of problems (participant 36, unscreened, delivered < 1 year). |
| **Empowered attitudes** | It is not really hard. Nothing is hard for real. If you put your mind to doing it, you’ll do it (participant 1, screened, delivered > 1 year).  With this pregnancy I actually went to see a nutritionist early on because I said I’m not going to get gestational diabetes… I started off really, really early trying to eat healthy (participant 12, screened, delivered > 1 year).  If you’re not happy, you can’t make nobody else happy. If you’re not healthy, you can’t expect to help anyone, you know, so I guess getting the number one thing is me getting to a place of stability in all things (participant 28, unscreened, delivered < 1 year).  People are really trying to change for the better with their health and I do see more people exercising, so that’s a change that’s coming (participant 31, screened, delivered > 1 year).  I didn’t want nothing to go wrong with myself. So that’s why I came in (participant 35, screened, delivered > 1 year).  I’m pretty much like a robot. I just go in with the flow and take care of what I got to take care (participant 52, unscreened, delivered < 1 year).  I was really anxious about knowing now…am I diabetic?... Or was it just, you know, a pregnancy thing…that was high on my list (participant 53, screened, delivered < 1 year).  If they tell you the steps and everything, take, take note of it and proceed with everything that they have given you. So now I see that I have to take better care of myself because if I don’t do it, who else is going to do it (participant 48, unscreened, delivered > 1 year)?  There is a possibility that I may end up getting it so I just basically keep asking my doctor to keep constantly checking me to make sure that I don’t end up entering into diabetes….Basically just if you feel that something is wrong with your body to basically go to the doctor and check… Don’t wait  the last minute  try to check (participant 29, screened, delivered > 1 year).  I think it was more so of like [not] being another number like another person in the Black community with diabetes…Because that is something that is high beside high blood pressure, that is something that kind of wipes... Our race away, you know (participant 45, screened, delivered < 1 year). |
| **Healthy food and exercise patterns** | I got away from juice. I used to drink a lot of juice and I realized that mostly they had a lot of sugar and I started drinking a lot of water (participant 2, screened, delivered > 1 year).  I would notice like processed food. If I ate like fast food like or something that was packaged, it would make my blood sugar go up but when I spent time and cooked a real meal, my blood sugar would be normal (participant 28, unscreened, delivered < 1 year).  I actually lost between my daughter and my son…about 67 pounds because…I just cut out soda. I just start drinking water, just ate a lot of fruits and vegetables, a lot of fish (participant 12, screened, delivered > 1 year).  It’s important for me, very important for me to be able to go to the gym, be able to ah get my stress level down, meditate (participant 10, unscreened, delivered > 1 year).  I cooked maybe 3 days’ worth of meals, breakfast, lunch and dinner in little containers in the refrigerator. So…all of it is already set up so I can just go and warm it up, still spending a lot of time with him but, you know, make sure that I’m not trying to go over to McDonald’s (participant 3, screened, delivered < 1 year).  I do not do breads, pastas and rice, like I do not feel like I am strong enough to proportion it out so I just do not do it at all (participant 32, unscreened, delivered > 1 year).  I make sure my kids they eat way better than me. They eat a lot of veggies, they drink milk. So I make sure that they don’t have that problem. While growing up (participant 46, screened, delivered > 1 year).  It motivates me more, you know, I have to work out every day. If I don’t, I just feel lazy, don’t feel like doing nothing (participant 4, screened, delivered > 1 year). |
